# Nuclear F-actin and Lamin A antagonistically modulate nuclear shape

**DOI:** 10.1242/jcs.259692

**Published:** 2022-07-04

**Authors:** Sampada Mishra, Daniel L. Levy

**Affiliations:** Department of Molecular Biology, University of Wyoming, Laramie, WY 82071, USA

**Keywords:** HeLa, Lamin A, *Xenopus*, Actin, Formins, Nuclear shape

## Abstract

Nuclear shape influences cell migration, gene expression and cell cycle progression, and is altered in disease states like laminopathies and cancer. What factors and forces determine nuclear shape? We find that nuclei assembled in *Xenopus* egg extracts in the presence of dynamic F-actin exhibit a striking bilobed nuclear morphology with distinct membrane compositions in the two lobes and accumulation of F-actin at the inner nuclear envelope. The addition of Lamin A (encoded by *lmna*), which is absent from *Xenopus* eggs, results in rounder nuclei, suggesting that opposing nuclear F-actin and Lamin A forces contribute to the regulation of nuclear shape. Nuclear F-actin also promotes altered nuclear shape in Lamin A-knockdown HeLa cells and, in both systems, abnormal nuclear shape is driven by formins and not Arp2/3 or myosin. Although the underlying mechanisms might differ in *Xenopus* and HeLa cells, we propose that nuclear F-actin filaments nucleated by formins impart outward forces that lead to altered nuclear morphology unless Lamin A is present. Targeting nuclear actin dynamics might represent a novel approach to rescuing disease-associated defects in nuclear shape.

## Teaser

**Summary:** Formin-nucleated F-actin and Lamin A antagonistically modulate nuclear shape in *Xenopus* egg extracts and HeLa cells, which might be relevant in diseases with altered nuclear morphology.

## INTRODUCTION

How nuclear shape influences nuclear function is an important question necessitating a complete understanding of the mechanisms that regulate nuclear shape. Although nuclei are often roughly spherical, there is significant variation in nuclear shape among various cell types and between different species, ranging from ovoid to multilobed (Skinner and Johnson, 2017; Webster et al., 2009). Nuclear shape deviations occur during aging and are characteristic of disease states like laminopathies and cancer (Bell and Lammerding, 2016; Diamond et al., 1982; Goldman et al., 2004; Scaffidi and Misteli, 2006; Skinner and Johnson, 2017; Steele-Stallard et al., 2018; Sullivan et al., 1999; Webster et al., 2009; Zink et al., 2004). How nuclear shape influences cell function is not completely understood, although effects on cell migration, gene expression and cell cycle progression have been implicated (Aureille et al., 2019; Hoffmann et al., 2007; Khatau et al., 2009; Thomas et al., 2002).

Nuclear shape is determined by factors and forces both inside and outside the nucleus. Relevant intranuclear factors include the nuclear lamina, nucleoporins and chromatin modifications and structure, whereas cytoplasmic influences include lipid metabolism and trafficking, the endoplasmic reticulum, mechanotransduction and the cytoplasmic F-actin cap (Aureille et al., 2019; Jevtić et al., 2014; Khatau et al., 2009; Kim et al., 2017; Siniossoglou, 2009; Stephens et al., 2019; Walters et al., 2012; Webster et al., 2010; Worman, 2012). The nuclear lamina, composed of a meshwork of lamin intermediate filament proteins, confers structural integrity to the nuclear envelope (Burke and Stewart, 2013; Dittmer and Misteli, 2011; Gerace and Huber, 2012). B-type lamins are present in most cell types, whereas in early embryonic and stem cells, A-type lamins are absent or expressed at very low levels (Dechat et al., 2010; Dittmer and Misteli, 2011). In addition to contributing to the rigid structure of the nucleus, A-type lamins also regulate signaling pathways, mechanosensing, chromatin organization, gene expression, genomic stability and DNA damage repair (Andrés and González, 2009; Dahl et al., 2008; Dubik and Mai, 2020; Lammerding et al., 2006; Schäpe et al., 2009). Of note, Lamin A (encoded by *lmna*) mutations and changes in Lamin A/C expression levels are implicated in many cancers as well as laminopathies associated with progeria, cardiomyopathy and muscular dystrophy (Andrés and González, 2009; Bell and Lammerding, 2016; Dubik and Mai, 2020). A defining feature of many of these diseases is altered nuclear morphology, yet questions remain about how Lamin A dysfunction leads to changes in nuclear shape, changes that might directly impact cellular physiology.

Actin is one of the most abundant proteins in cells and polymerizes to form F-actin filaments. Actin-based structures in the cytoplasm include cortical actin, stress fibers and the perinuclear actin cap, whereas actin within the nucleus is present as monomeric G-actin as well as nuclear actin polymers and rods (Davidson and Cadot, 2021; Kelpsch and Tootle, 2018; Kita et al., 2019; Procter et al., 2020; Roeles and Tsiavaliaris, 2019; Svitkina, 2020; Tojkander et al., 2012; Wang et al., 2019; Wineland et al., 2018). A key cytoplasmic determinant of nuclear shape in adherent cells is the perinuclear F-actin cap, a specialized contractile actomyosin structure connected to the nucleus through linker of nucleoskeleton and cytoskeleton (LINC) complexes (Khatau et al., 2009). On the contrary, nuclear actin regulates diverse processes such as transcription, chromatin remodeling, DNA replication and DNA damage repair (Hurst et al., 2019; Kelpsch and Tootle, 2018; Krauss et al., 2003; Lamm et al., 2020; Okuno et al., 2020; Parisis et al., 2017; Wesolowska et al., 2020). Of particular interest, nuclear F-actin drives nuclear expansion and promotes the mechanical integrity of the nucleus (Baarlink et al., 2017; Bohnsack et al., 2006). If and how dynamic F-actin within the nucleus influences nuclear shape are open questions.

Because a wide variety of different factors with pleiotropic functions impinge on nuclear shape, we sought a simplified system to investigate the involvement of nuclear F-actin in nuclear shape regulation. *Xenopus* egg extracts support *de novo* nuclear assembly *in vitro* and lack endogenous Lamin A, containing predominantly the embryonic Lamin B3 (*lmnb3* in *Xenopus*) (Gareiss et al., 2005; Good and Heald, 2018; Levy and Heald, 2010; Nmezi et al., 2019; Peter and Stick, 2008). The perinuclear F-actin cap and cell cortex are absent, eliminating potentially confounding effects from these cytoplasmic actin structures. Furthermore, the extract is transcriptionally inert, allowing us to rule out possible effects of actin on transcription (Newport and Kirschner, 1982b; Wang and Shechter, 2016). In contrast to traditional *Xenopus* egg extracts supplemented with the actin-depolymerizing drug cytochalasin B (Good and Heald, 2018; Murray, 1991), we used extracts lacking cytochalasin B, and thus containing intact F-actin, in order to investigate how dynamic F-actin influences nuclear shape (Field et al., 2017). Using this approach, we demonstrate that formin-mediated F-actin nucleation within the nucleus influences nuclear shape. Complementary data from *Xenopus* egg extracts and mammalian cells support a model whereby nuclear shape is determined by the antagonistic action between Lamin A and nuclear F-actin.

## RESULTS

### F-actin induces bilobed nuclear morphology in *Xenopus* egg extracts

**Fig. 1.**
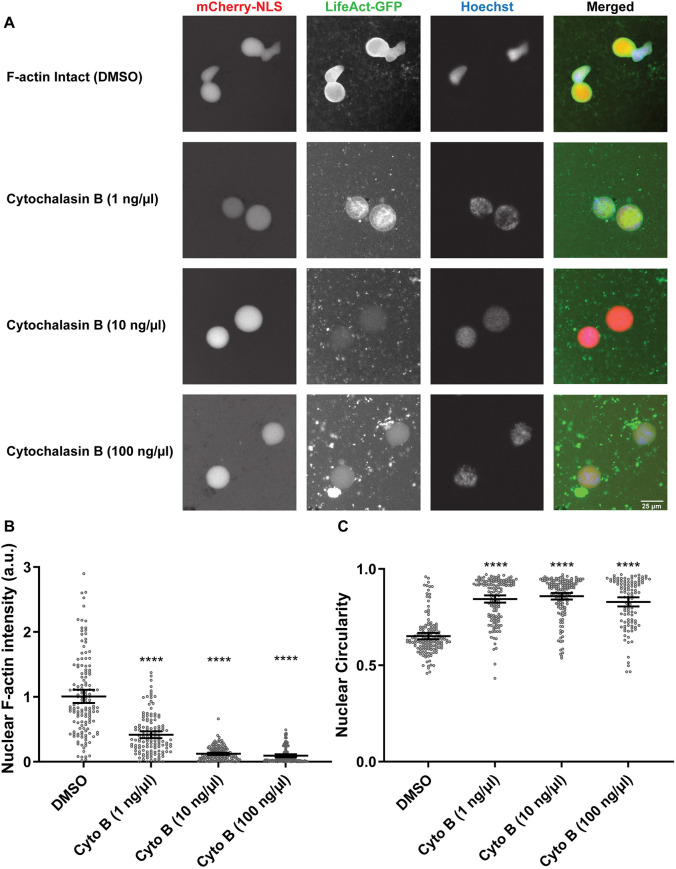
**F-actin induces bilobed nuclei in *Xenopus* egg extracts.** (A) F-actin-intact extracts were supplemented with either DMSO or different concentrations of cytochalasin B (Cyto B). After 90 min of nuclear assembly, nuclei were visualized by the import of mCherry–NLS, F-actin was visualized with LifeAct–GFP and DNA was visualized with Hoechst 33342. (B) Total nuclear F-actin intensity was measured based on LifeAct–GFP signal intensity (Pelletier et al., 2020), normalized to DMSO controls. Based on two independent experiments, the nucleus numbers quantified were: DMSO, *n*=141; Cyto B (1 ng/µl), *n*=133; Cyto B (10 ng/µl), *n*=141; Cyto B (100 ng/µl), *n*=108. (C) Nuclear circularity was quantified. Based on two independent experiments, the nucleus numbers quantified were: DMSO, *n*=141; Cyto B (1 ng/µl), *n*=137; Cyto B (10 ng/µl), *n*=141; Cyto B (100 ng/µl), *n*=108. Images were obtained by widefield microscopy and are representative of two experiments. Mean values and 95% c.i. error bars are shown. Nonparametric Kruskal–Wallis tests were performed, showing statistical significance relative to DMSO controls. a.u., arbitrary units. ****P≤0.0001.

**Fig. 2.**
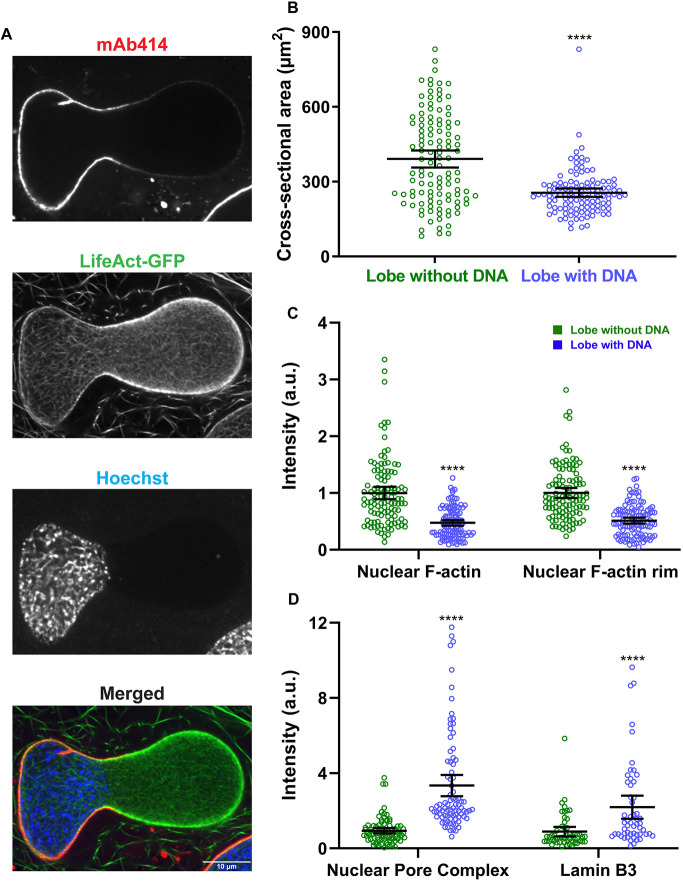
**The nuclear envelope is structurally heterogeneous in bilobed nuclei.** (A) After 90 min of nuclear assembly in F-actin-intact extracts, nuclei were stained with mAb414 (to label NPCs), LifeAct–GFP (F-actin) and Hoechst 33342 (DNA) and imaged by confocal microscopy. In some cases, F-actin was labeled with Alexa Fluor 488 phalloidin. A bilobed nucleus is shown (representative of four experiments). (B) Nuclear cross-sectional area was quantified based on F-actin staining for the lobes with and without Hoechst 33342-stained DNA (*n*=113 nuclei, five independent experiments) using widefield microscopy. (C) Total nuclear F-actin and F-actin localized to the nuclear rim were quantified (*n*=113 nuclei, five independent experiments) using widefield microscopy, in each case normalized to the lobe without Hoechst 33342-stained DNA. (D) NPC intensity in the two lobes was quantified based on mAb414 staining (*n*=85 nuclei, four independent experiments) using widefield microscopy. To quantify Lamin B3 intensity in the two lobes, extracts were supplemented with GFP–Lamin B3 and imaging performed by confocal microscopy (*n*=54 nuclei). In both cases, intensity values were normalized to the lobe without Hoechst 33342-stained DNA. Mean values and 95% c.i. error bars are shown. Nonparametric Mann–Whitney tests were performed, showing statistical significance relative to the lobe without Hoechst 33342-stained DNA. a.u., arbitrary units. ****P≤0.0001.

Nuclei are roughly spherical when assembled in *Xenopus* egg extracts in the presence of the actin-depolymerizing drug cytochalasin B. To determine whether F-actin affects nuclear shape in extracts, we assembled nuclei in extracts devoid of cytochalasin B containing intact F-actin filaments (hereby referred to as F-actin-intact extracts). Nuclear shape was measured in terms of circularity, wherein perfectly round nuclei have a circularity of one and values less than one indicate nuclei with more abnormal shapes. Strikingly, nuclei were bilobed in shape with reduced circularity in the presence of F-actin (Fig. 1A,C). Live imaging with the F-actin probe LifeAct–GFP showed that F-actin was present throughout the nucleoplasm as well as being enriched at the nuclear periphery (Fig. 1A, Fig. 2A). Bilobed nuclei were still observed in the absence of LifeAct–GFP, eliminating concerns that the LifeAct–GFP probe itself might influence nuclear morphology (Fig. S1). Cytochalasin B addition resulted in rounder nuclei (27–32% increase in circularity) with a concomitant decrease in total nuclear and nucleoplasmic F-actin intensity (Fig. 1; Fig. S2A), without affecting the total amount of nuclear actin (Fig. S2D,E). Some nucleoplasmic F-actin filaments were still visible with low concentrations of cytochalasin B, suggesting that nucleoplasmic F-actin might be more resistant to cytochalasin B-mediated depolymerization than F-actin localized at the nuclear rim. In addition, nuclei were larger in the presence of F-actin (Fig. S2B,C), consistent with previous observations in mammalian cells (Baarlink et al., 2017).

Hoechst 33342 staining of bilobed nuclei assembled in F-actin-intact extracts revealed that the DNA was generally localized to one of the two lobes, whereas it was more homogenously distributed in the absence of F-actin (Fig. 1A). To gain further insight into bilobe formation, we performed live imaging of nuclear growth in F-actin-intact extracts (Movie 1). Although initially round, nuclei eventually began to bleb with the size of the newly formed lobe increasing over time. Interestingly, Hoechst 33342-stained DNA remained localized in the original lobe and was mostly excluded from the new lobe, which grew more rapidly than the original lobe. Time-lapse imaging thus suggested that bilobe formation results from the growth of a DNA-free bleb after initial nucleus formation. We also performed live imaging of nuclear F-actin with LifeAct–GFP (Movies 2 and 3). Soon after nuclear assembly, F-actin filaments were apparent in the nucleoplasm and were also slightly enriched at the nuclear periphery. Upon further nuclear growth and coinciding with bilobe formation, the F-actin signal at the nuclear envelope became stronger, particularly in the lobe lacking DNA. Thus, there is a correlation between bilobe formation and enrichment of F-actin at the nuclear periphery.

### Bilobed nuclei exhibit intranuclear actin at the nuclear rim and heterogenous nuclear envelopes

To further characterize bilobed nuclei at later stages of growth, we compared the abundance of different proteins in the lobes with and without DNA. The lobe without DNA was larger in size with higher nuclear and rim-localized F-actin signals compared to the lobe with DNA (Fig. 2A–C). In contrast, nuclear pore complexes (NPCs) and Lamin B3 were more prevalent in the lobe containing the DNA, perhaps through a DNA-tethering effect (Fig. 2A,D; Fig. S1).

**Fig. 3.**
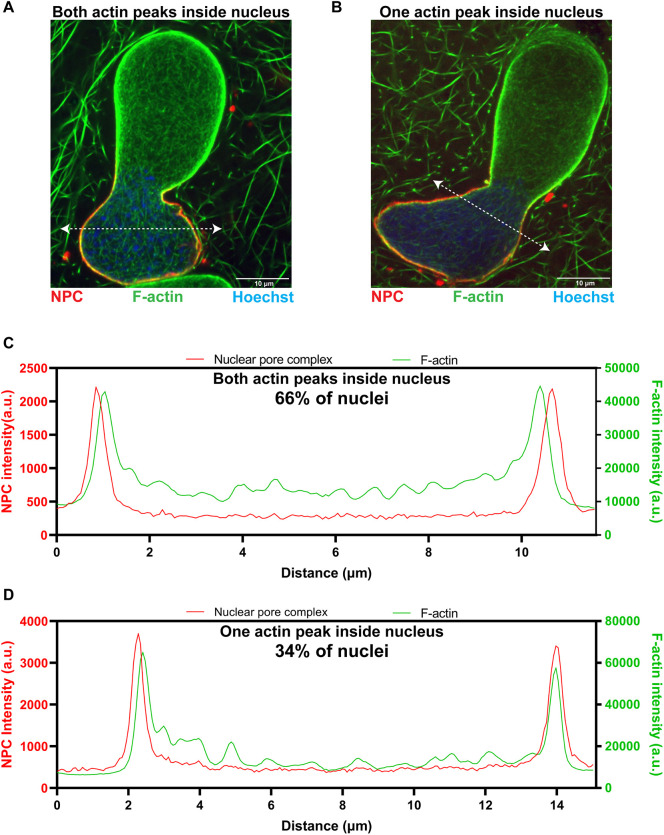
**Intranuclear F-actin is enriched at the nuclear rim in bilobed nuclei.** (A,B) Bilobed nuclei formed in F-actin-intact *Xenopus* egg extracts were imaged with mAb414 (NPCs, red), LifeAct–GFP (F-actin, green) and Hoechst 33342 (DNA, blue) using confocal microscopy. Intensity line scans (dotted white lines) were used to determine where F-actin localizes relative to the NE, determined by NPC staining. Representative nuclei in which both F-actin peaks are intranuclear with respect to the NPC peaks (A) and in which one actin peak is intranuclear with respect to the NPC peak and the other actin peak colocalizes with the NPC peak (B) are shown. (C) Intensity measurements for the line scan shown in A. (D) Intensity measurements for the line scan shown in B. In total, 65 nuclei were analyzed as shown in C and D from two independent experiments. a.u., arbitrary units.

Because the F-actin signal at the nuclear rim was more intense in the larger lobe, this suggested that F-actin might be generating force from within the nucleus to drive growth of the larger lobe. To determine whether this rim-localized pool of F-actin was inside or outside of the nucleus, we co-stained nuclei for F-actin and NPCs and performed line scans across nuclei, measuring intensities at opposing nuclear envelope (NE) regions (Fig. 3). For the majority of the nuclei (66%), both F-actin peaks were intranuclear with respect to the NPC peaks and, for the remaining nuclei (34%), one actin peak was intranuclear and the other co-localized with NPCs (Fig. 3C,D). These data show that F-actin enriched at the nuclear envelope is indeed intranuclear, appropriately positioned to potentially generate outward pushing forces against the nuclear envelope.

### Lamin A addition partially rescues the bilobed nuclear morphology

**Fig. 4.**
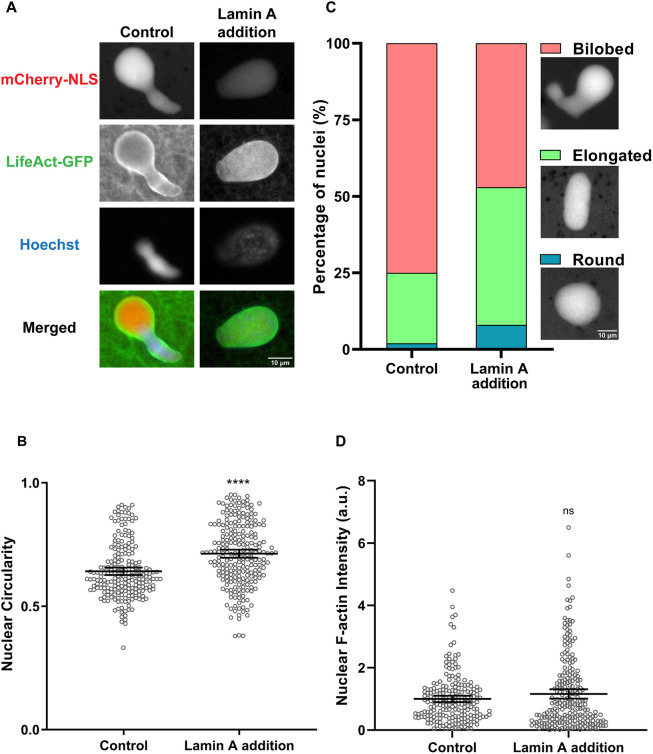
**Addition of Lamin A to F-actin-intact extracts partially rescues the bilobed nuclear phenotype.** (A) F-actin-intact extracts were supplemented with 280 nM recombinant Lamin A and nuclei were imaged after 90 min of nuclear assembly. Widefield images (representative of four experiments) are shown for a control nucleus (circularity 0.57) and a nucleus from Lamin A-supplemented extracts (circularity 0.85). (B) Nuclear circularity was quantified from four independent experiments for 212 control nuclei and 234 nuclei from Lamin-A supplemented extracts. (C) For the data presented in B, nuclei were characterized as bilobed (circularity <0.7), elongated (circularity 0.7-0.9) or round (circularity >0.9), and representative nuclei importing mCherry–NLS are shown. (D) Total nuclear F-actin was measured based on LifeAct–GFP staining and normalized to the control for 212 control nuclei and 237 nuclei from Lamin A-supplemented extracts from four independent experiments. Mean values and 95% c.i. error bars are shown. Nonparametric Mann–Whitney tests were performed, showing statistical significance relative to controls. a.u., arbitrary units. ns, not significant; ****P≤0.0001.

*Xenopus* egg extracts lack endogenous Lamin A/C (Gareiss et al., 2005; Peter and Stick, 2008), which is known to influence nuclear rigidity (Lammerding et al., 2006; Schäpe et al., 2009). We hypothesized that F-actin might induce nuclear bilobes in egg extracts due to an absence of bleb-resisting forces, such as those provided by Lamin A/C. To test this idea, we examined nuclear shape in F-actin-intact extracts supplemented with recombinant Lamin A, which properly incorporates into the NE (Fig. S3C) (Jevtić et al., 2015). Interestingly, Lamin A addition resulted in nuclei that were rounder or with an intermediate elongated phenotype (Fig. 4A), as illustrated by an increase in the average nuclear circularity by 11% (Fig. 4B). Whereas 75% of the control nuclei were bilobed (circularity <0.7), this percentage decreased to 47% upon Lamin A addition (Fig. 4C). Furthermore, Lamin A addition increased the percentage of intermediate elongated nuclei (circularity between 0.7 and 0.9) from 23% to 45% and the percentage of round nuclei (circularity >0.9) from 2% to 8%. We verified that this partial rescue of the bilobed nuclear morphology by Lamin A was not due to a reduction in total nuclear or rim-localized F-actin (Fig. 4D; Fig. S3A). These data suggest that nuclear F-actin and Lamin A might oppose each other to regulate nuclear shape.

### Nuclear F-actin affects nuclear shape in mammalian cells

**Fig. 5.**
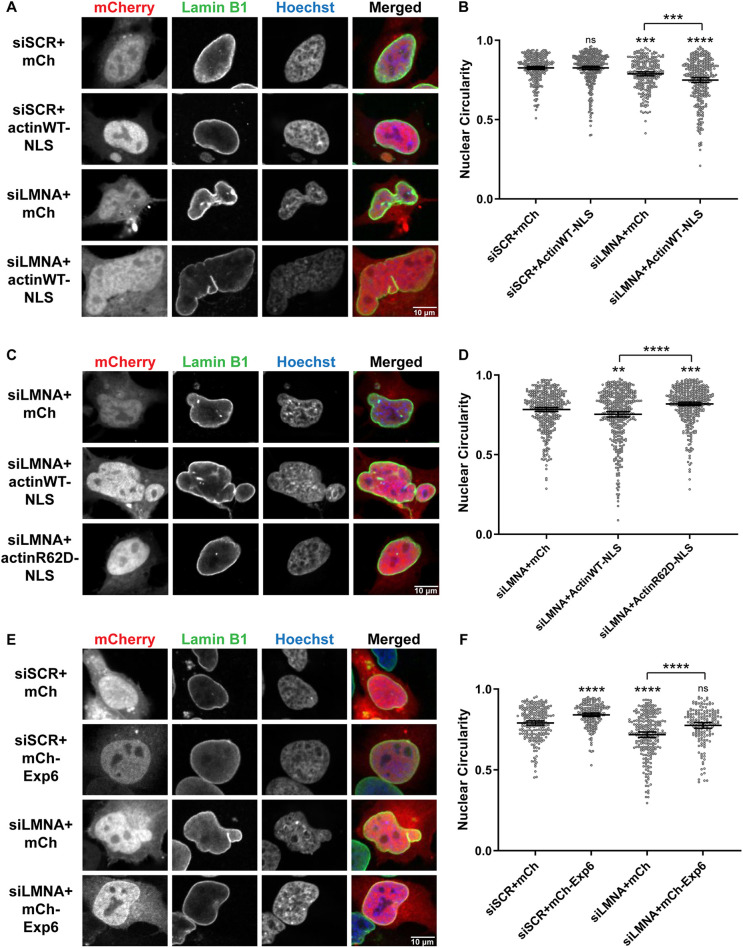
**Dynamic nuclear F-actin alters nuclear shape in HeLa cells.** HeLa cells were transfected with the indicated plasmids: mCh (pEmCherry-C2), actinWT–NLS (actin-3×NLS P2A mCherry), actinR62D–NLS (pmCherry-C1 R62D actin-3×NLS P2A) and mCh–Exp6 (pcDNA3.1-mCherry-Exp6). For knockdowns, cells were transfected with a scrambled control (siSCR) or siRNA targeted against Lamin A/C (siLMNA). Fixed cells were stained for Lamin B1 (green) and DNA (Hoechst 33342, blue). Only transfected cells expressing mCherry were quantified. (A) Representative images are shown. (B) Nuclear circularity measurements for A. Based on three independent experiments, the nucleus numbers quantified were: siSCR+mCh (*n*=283), siSCR+actinWT–NLS (*n*=357), siLMNA+mCh (*n*=244) and siLMNA+actinWT–NLS (*n*=332). (C) Representative images are shown. (D) Nuclear circularity measurements for C. Based on four independent experiments, the nucleus numbers quantified were: siLMNA+mCh (*n*=382), siLMNA+actinWT–NLS (*n*=412) and siLMNA+actinR62D–NLS (*n*=443). (E) Representative images are shown. (F) Nuclear circularity measurements for E. Based on two independent experiments, the nucleus numbers quantified were: siSCR+mCh (*n*=223), siSCR+mCh–Exp6 (*n*=195), siLMNA+mCh (*n*=255) and siLMNA+mCh–Exp6 (*n*=165). Images were obtained by confocal microscopy. Mean values and 95% c.i. error bars are shown. One-way ANOVA with multiple comparisons and post-hoc Tukey tests were performed, showing statistical significance relative to controls. ns, not significant; **P≤0.01; ***P≤0.001; ****P≤0.0001.

Up until this point, our results were obtained using an *in vitro* system that lacks a cell cortex. We next performed experiments to determine if an interplay between Lamin A and nuclear F-actin regulates nuclear shape in HeLa cells, in which nuclear actin has been observed (Baarlink et al., 2013; Chatzifrangkeskou et al., 2019; McDonald et al., 2006; Serebryannyy et al., 2016). Nuclei in HeLa cells are usually ovoid in shape (Fig. 5A,E, top panels) and not as round as nuclei formed in cytochalasin B-treated egg extracts (Fig. 1A). Lamin A knockdown (Fig. S4) resulted in nuclei that were less round (5% decrease in circularity) and more lobulated (Fig. 5A,B), consistent with previous studies (Libotte et al., 2005; Song et al., 2016; Tariq et al., 2017). To increase nuclear actin levels, we transfected cells with a nuclear-targeted actin construct that includes an mCherry reporter separated from the actin–nuclear localization signal (NLS) construct by a P2A cleavage site (Belin et al., 2015). Interestingly, overexpressing nuclear-targeted actin in Lamin A-knockdown cells led to a further 9% decrease in circularity and exacerbated the lobulation phenotype (Fig. 5A,B), with some instances of multilobed nuclei (Fig. S5). Whereas only 5% of Lamin A-knockdown cells exhibited aberrant nuclei with circularity values below 0.6, this percentage increased to 16% when nuclear-targeted actin was overexpressed (Fig. 5B; Fig. S5B). Interestingly, 60% of these aberrant nuclei exhibited heterogeneous nuclear envelopes in which regions with reduced Lamin B1 (*lmnb1*) and NPC staining frequently coincided with regions of less DNA (Fig. S6A), similar to what we observed in F-actin-intact *Xenopus* extracts (Fig. 2). These data indicate that nuclear actin can exacerbate Lamin A-associated nuclear morphology defects. We note that overexpressing nuclear-targeted actin in the presence of Lamin A did not have a significant effect on nuclear morphology (Fig. 5A,B), possibly due to already high nuclear actin levels in HeLa cells and/or nuclear F-actin forces being resisted by Lamin A.

To visualize nuclear F-actin dynamics in Lamin A-knockdown HeLa cells overexpressing nuclear actin, we performed live imaging with a nuclear-targeted GFP-tagged actin chromobody probe (nAC–GFP) (Baarlink et al., 2017). After post-mitotic nuclear formation, F-actin was mostly present in the nucleoplasm as rods and filaments (Movies 4 and 5) and, after some time, F-actin accumulated at the nuclear rim in a subset of these cells (36%) (Fig. S6B; Movies 4–7). This localization is reminiscent of what we observed in *Xenopus* egg extracts (Movies 2 and 3) but might also correspond to actin cable bundles curving along the inner rim of the nucleus. In the majority of these nuclei (70%), rim-localized F-actin coincided with regions of reduced Lamin B1 and DNA staining (Fig. S6B).

To determine when in the cell cycle aberrant nuclear morphologies appeared in Lamin A-knockdown cells overexpressing nuclear actin, we performed live imaging of H2B–GFP in synchronized HeLa cells (Fig. S7). In the majority of cases (45%), round nuclei formed immediately after mitosis and subsequently underwent shape changes later in interphase (S/G2). In 20% of cells, nuclei exhibited aberrant shapes immediately after mitosis in the absence of any obvious mitotic defects (G1). A small fraction of cells (10%) exhibited mitotic defects (e.g. abnormal metaphase plate, lagging chromosomes, micronuclei, delayed or failed cytokinesis); however, this number was similar to the frequency of mitotic defects observed in control cells (8%). Thus, nuclear shape changes predominantly occur during interphase and not as a result of mitotic defects.

To determine whether F-actin filaments within the nucleus induce nuclear lobulation, we utilized a nuclear-targeted non-polymerizable mutant of actin (actinR62D–NLS). Whereas wild-type (WT) actin-NLS decreased nuclear circularity in Lamin A-knockdown cells, nuclear lobulation was not induced by actinR62D–NLS expression and the nuclear circularity value in actinR62D–NLS-expressing cells was 9% larger compared to WT actin–NLS-expressing cells, indicating that polymerizable nuclear F-actin is required to alter nuclear shape (Fig. 5C,D). In fact, actinR62D–NLS expression actually increased nuclear circularity by 5% as compared to Lamin A-knockdown alone, perhaps through a dominant-negative effect on nuclear F-actin structure. These data suggest that nuclear F-actin, rather than G-actin, drives altered nuclear shape.

To test whether the altered nuclear morphology observed in Lamin A-knockdown cells depends on nuclear actin, we induced nuclear export of actin by Exportin-6 (*xpo6*) overexpression (Stuven et al., 2003). Decreasing nuclear actin levels in Lamin A-knockdown cells resulted in rounder nuclei with an average circularity value comparable to control cells, indicating complete rescue of the nuclear morphology defect caused by Lamin A depletion (Fig. 5E,F). This result indicates that Lamin A-associated nuclear shape changes likely depend on nuclear actin. Furthermore, Exportin-6 overexpression in control cells also resulted in rounder nuclei (6% increase in circularity), suggesting that the ovoid nuclear shape in HeLa cells is at least partially due to nuclear actin (Fig. 5E,F). Taken together, these data show that nuclear F-actin and Lamin A oppose each other to regulate nuclear shape in HeLa cells, consistent with our observations in *Xenopus* extracts.

### Formins nucleate F-actin in the nucleus to regulate nuclear shape

**Fig. 6.**
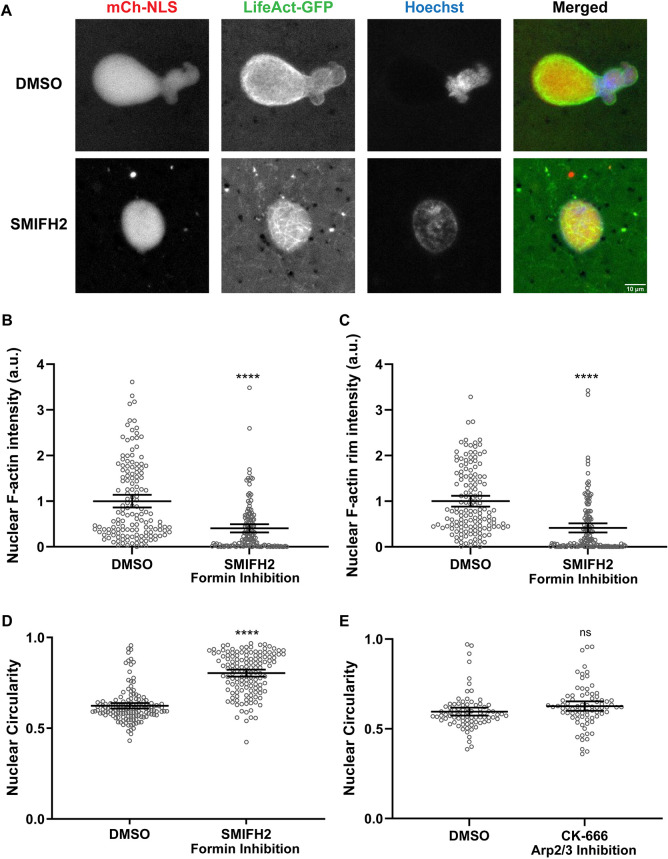
**Formin inhibition rescues bilobed nuclear morphology in *Xenopus* egg extracts.** (A–D) Experiments were performed as in Fig. 1 with F-actin-intact extracts except that DMSO or 500 µM SMIFH2 was added after 45 min of nuclear assembly. Based on three independent experiments, 144 DMSO-treated nuclei and 137 SMIFH2-treated nuclei were quantified. (A) Representative images from live imaging are shown. (B) Total nuclear F-actin intensity was quantified based on LifeAct–GFP staining and normalized to the DMSO control. (C) F-actin intensity at the nuclear rim was quantified based on LifeAct–GFP staining and normalized to the DMSO control. (D) Nuclear circularity was quantified. (E) Experiments were performed as in Fig. 1 with F-actin-intact extracts except that DMSO or 500 µM CK-666 was added after 45 min of nuclear assembly. Based on three independent experiments, nuclear circularity was quantified for 83 DMSO-treated nuclei and 79 CK-666-treated nuclei. Mean values and 95% c.i. error bars are shown. Nonparametric Mann–Whitney tests were performed on the SMIFH2 data. two-tailed unpaired Student's *t*-tests were performed on the CK-666 data. Statistical significance is shown relative to DMSO controls. a.u., arbitrary units. ns, not significant; ****P≤0.0001.

To rapidly screen for F-actin regulators that might affect nuclear shape, we returned to *Xenopus* egg extracts, testing various small molecule inhibitors. Inhibiting formins with the formin inhibitor SMIFH2 in F-actin-intact extracts reduced the staining intensity of nucleoplasmic and rim-localized F-actin (Fig. 6A–C; Fig. S3B), suggesting that much of the nuclear F-actin in *Xenopus* extracts is composed of linear, formin-nucleated actin filaments. Comparable to cytochalasin B treatment (Fig. 1C), formin inhibition resulted in rounder nuclei (29% increase in circularity) and fewer bilobes (Fig. 6A,D), implicating nuclear formins in the regulation of nuclear shape. However, nuclear shape was unaltered upon inhibiting Arp2/3 or myosin (Fig. 6E; Fig. S3D–F), suggesting that branched actin filaments and actomyosin-mediated forces are not required for bilobe formation.

**Fig. 7.**
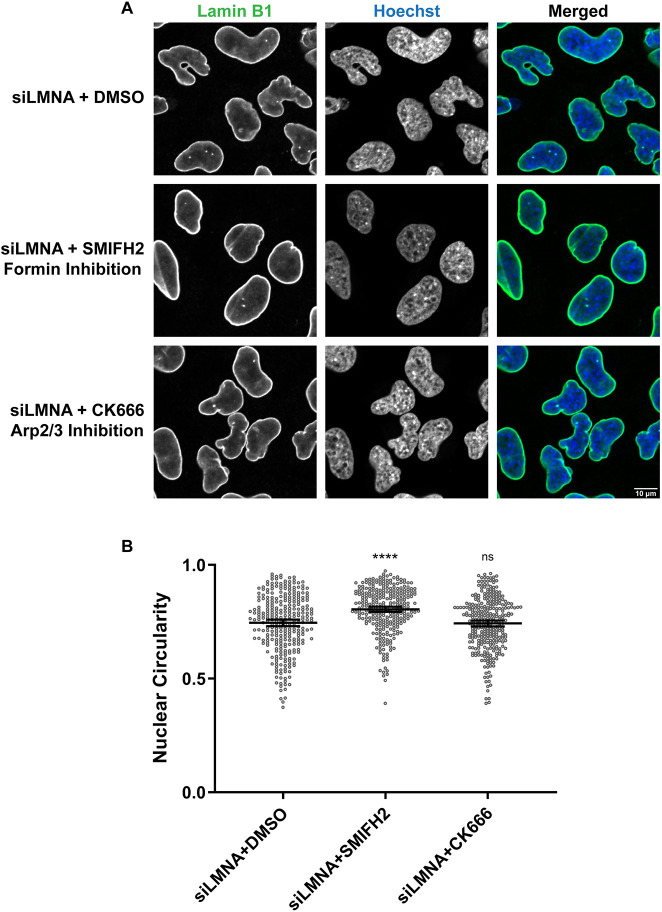
**Formin inhibition rescues nuclear shape defects caused by Lamin A depletion in HeLa cells.** Lamin A/C-knockdown HeLa cells were treated with either 25 µM SMIFH2, 100 µM CK-666 or DMSO as a control for 1 h. Fixed cells were stained for Lamin B1 (green) and DNA (Hoechst 33342, blue). (A) Representative images are shown. (B) Based on three independent experiments, nuclear circularity was quantified for 293 siLMNA+DMSO nuclei, 299 siLMNA+SMIFH2 nuclei and 305 siLMNA+CK-666 nuclei. Images were obtained by confocal microscopy. Mean values and 95% c.i. error bars are shown. Nonparametric Kruskal–Wallis tests were performed, showing statistical significance relative to DMSO controls. ns, not significant; ****P≤0.0001.

To test the involvement of actin nucleators in nuclear shape regulation in HeLa cells, we treated Lamin A-knockdown cells with the Arp2/3 inhibitor CK666 or the formin inhibitor SMIFH2, using low concentrations and a short 1 h incubation to minimize cytotoxic effects (Isogai et al., 2015). Nuclear circularity was increased by 8% upon formin inhibition whereas Arp2/3 inhibition had no effect (Fig. 7). Consistent with the results using *Xenopus* extracts, formins appear to be the predominant actin regulators responsible for altering nuclear shape in the absence of Lamin A. The finding that inhibiting formins for only 1 h was sufficient to largely rescue the nuclear shape defects resulting from Lamin A knockdown suggests that dynamic F-actin is required to maintain abnormal nuclear shape and that passage through mitosis is not required to rescue nuclear morphology.

## DISCUSSION

Taken together, our data from *Xenopus* extracts and HeLa cells suggest that nuclear formins contribute to F-actin nucleation in the nucleus, generating nucleoplasmic actin filaments. We propose that F-actin accumulation at the nuclear rim occurs through the movement of nucleoplasmic filaments toward the nuclear envelope and/or by actin nucleation at the rim. In the absence of Lamin A, in F-actin-intact *Xenopus* extracts or in Lamin A-knockdown Hela cells for instance, nuclear F-actin can impart outward forces that lead to growth of nuclear lobes and altered nuclear morphology. However, in *Xenopus* extracts supplemented with Lamin A or in untreated HeLa cells, the force generated by nuclear F-actin is resisted by the presence of Lamin A, preventing the lobulation of nuclei and resulting in rounder nuclei. Thus, we propose that a balance of forces between nuclear F-actin and nuclear Lamin A is a key determinant of nuclear shape, although the underlying mechanisms might differ between *Xenopus* egg extracts and HeLa cells.

Single linear actin filaments, such as those nucleated by formins, can generate forces on the order of piconewtons (Footer et al., 2007; Kovar and Pollard, 2004). In the absence of counteracting forces, it is conceivable that a meshwork of actin filaments acting locally on the inner nuclear membrane could apply nanonewtons of force leading to deformation of the NE and bleb formation. Although we do not know whether force is being generated by actin filaments in the nucleoplasm and/or at the nuclear rim, F-actin acting at the rim is implicated by the fact that treatment with low concentrations of cytochalasin B (Fig. 1A) and formin inhibition (Fig. 6A) preferentially depolymerize rim-localized actin and cause nuclear rounding. The nuclear lamina is reversibly elastic when subjected to piconewton forces whereas nanonewtons of applied force cause lamina stiffening (Sapra et al., 2020). In the presence of Lamin A, the nuclear actin might exert nanonewton forces, in turn leading to lamina stiffening, which resists deformation of the NE and promotes rounder nuclei. It is worth noting that some cell types possess a cytoplasmic F-actin cap that lines the outside of the nucleus and influences nuclear shape. Although HeLa nuclei (Kim et al., 2013) and nuclei assembled in *Xenopus* extracts do not exhibit a defined actin cap, it will be important to consider the relative contributions of nuclear F-actin, Lamin A/C and the cytoplasmic F-actin cap in regulating nuclear morphology in other cell types.

Although nucleoplasmic actin filaments and rods are well-described in the literature, to what extent is intranuclear accumulation of F-actin at the NE a conserved feature of nuclear structure? In starfish oocytes, an F-actin shell forms at the NE to promote nuclear envelope breakdown (Bun et al., 2018; Mori et al., 2014; Wesolowska et al., 2020). Similar F-actin nuclear shells have been observed in a variety of other species including early sea urchin and sand dollar embryos, *Nematostella vectensis* and polychaete worms (Burkel et al., 2007; DuBuc et al., 2014; Jacobsohn, 1999). F-actin is also observed at the nuclear rim in *Xenopus laevis* oocytes (Bohnsack et al., 2006) and mouse oocytes (Scheffler et al., 2022), as well as in mesenchymal stem cells (Sankaran et al., 2020) and a variety of cultured mammalian cell lines (Baarlink et al., 2013). The prominence of this structure might depend on cell type, species, developmental stage and/or disease state. For example, accumulation of actin at the intranuclear rim might be a characteristic of nuclei in oocytes and early-stage embryos, perhaps due to their large nuclear sizes. This might explain why nuclear rim-localized actin is more prominent in large egg extract nuclei than in smaller HeLa cell nuclei, providing evidence that the extract system recapitulates a normal feature of oocyte nuclear structure. Thus F-actin at the intranuclear rim is likely physiologically relevant, at least in the context of early development, underscoring the need to examine this structure in other cell types.

What determines the site of nuclear lobe formation? Lamin B1 is known to preferentially localize to low curvature membrane domains and to be less abundant in regions in which membrane curvature is high (Nmezi et al., 2019). Nuclei assembled in *Xenopus* extracts are not perfectly spherical, so it is possible that less Lamin B3 accumulates at regions of higher curvature. These areas of the NE with reduced Lamin B3 might be more susceptible to actin-mediated force deformation and nuclear lobulation. Alternatively, formins might be preferentially recruited to NE regions that are low in Lamin B3 and/or are more highly curved. Once lobe formation is initiated, F-actin would then drive continued growth of the lobe with reduced incorporation of new NPCs and Lamin B3.

Although we observed bilobed nuclei in F-actin-intact *Xenopus* egg extracts that lacked endogenous Lamin A/C, nuclei have been reported to be fairly spherical in early-stage *Xenopus* embryos also devoid of Lamin A/C (Gareiss et al., 2005; Levy and Heald, 2010; Peter and Stick, 2008). The first possible explanation is that Exportin-6 expression increases after fertilization (Peshkin et al., 2015), leading to reduced nuclear actin levels. Consistent with this idea, we showed that increasing Exportin-6 expression in Lamin A-knockdown HeLa cells led to rounder nuclei (Fig. 5E,F). Secondly, in embryos, there is an intact cell cortex that is absent from egg extracts, and cytoskeletal connections between the cell cortex and cytoplasm via LINC complexes might stabilize nuclear shape. Thirdly, early developing embryos have very fast cell cycles with short interphases with no gap phases (Newport and Kirschner, 1982a), which might not provide enough time for nuclear bilobe formation. Finally, factors other than Lamin A might stabilize nuclei against lobe formation. Future experiments will address the regulation of nuclear shape in the early embryo.

Misshapen nuclei formed in *Xenopus* extracts and HeLa cells frequently exhibited uneven distributions of NE proteins, for instance, regions of reduced DNA density often correlated with reduced staining for NPCs and lamins, reminiscent of nuclear blebs that form during confined cell migration (Denais et al., 2016; Raab et al., 2016). Similarly, Lamin A knockdown in mouse embryonic fibroblasts resulted in elongated nuclei with reduced Lamin B, Lap2 and NPCs at one pole (Sullivan et al., 1999), and nuclear blebs in progeroid fibroblasts expressing mutated Lamin A exhibited reduced staining for DNA, NPCs and Lamin B1 (Bercht Pfleghaar et al., 2015). What might account for this uneven distribution of lamins and NPCs across the NE? LEM-domain proteins like Lap2, emerin and Man1 interact with both the nuclear lamina and barrier-to-autointegration factor (BAF)-bound DNA, effectively tethering chromatin to the lamina (Shimi et al., 2004; Wilson and Foisner, 2010). Similarly, the nucleoporin ELYS binds chromatin via histone interactions (Zierhut et al., 2014). Lamins and NPCs might therefore be preferentially tethered to regions with the most DNA content through chromatin-mediated interactions. Another intriguing possibility is that upon nuclear import, lamins are preferentially incorporated into the region of the lamina close to importing NPCs. This might explain why NE regions containing fewer NPCs also show reduced lamin incorporation. As the nuclear lobe without DNA grows more rapidly than the DNA-containing lobe in F-actin-intact extracts, this system can be used to study uncoupling of nuclear growth and chromatin dynamics.

Nuclear actin has well-established roles in cancer, myopathies and neurodegeneration through its effects on chromatin remodeling, transcriptional regulation and DNA damage repair (Kelpsch and Tootle, 2018; Serebryannyy and de Lanerolle, 2020; Zahler, 2020). Because *Xenopus* egg extracts are transcriptionally inert (Newport and Kirschner, 1982b; Wang and Shechter, 2016), we have clearly defined a transcription-independent role for nuclear F-actin in modulating nuclear morphology. This finding might prompt future studies into how physical force exerted by nuclear F-actin contributes to disease, as opposed to the influence of G-actin on transcription.

Irregular nuclear shape and nuclear blebs are associated with various laminopathies, progerias and cancers (Bell and Lammerding, 2016; Diamond et al., 1982; Goldman et al., 2004; Scaffidi and Misteli, 2006; Skinner and Johnson, 2017; Steele-Stallard et al., 2018; Sullivan et al., 1999; Webster et al., 2009; Zink et al., 2004), and altered nuclear morphology in many cancers correlates with changes in lamin expression levels (Bell and Lammerding, 2016; Dubik and Mai, 2020). In these various diseases, lamin mutations or changes in lamin expression have been evoked as the underlying cause of irregular nuclear morphology. Although altered nuclear lamina structure might render these nuclei more susceptible to changes in nuclear shape, our data reveal that forces exerted by nuclear F-actin might in fact be the direct effectors of these nuclear shape alterations. For example, HeLa cells exhibit irregular nuclear shapes characteristic of cancer (Fig. 5A,E), and we largely corrected this nuclear shape defect by inducing export of nuclear actin (Fig. 5E,F) or by decreasing the amount of dynamic polymerizable nuclear actin (Fig. 5C,D). Thus, nuclear F-actin might be a key determinant of nuclear shape in laminopathies and cancer, and novel therapeutic approaches might be developed that target nuclear actin-related proteins and activities. Future studies will focus on the role of nuclear F-actin in regulating nuclear morphology in normal and diseased cells, work that promises to inform the functional significance of nuclear shape.

## MATERIALS AND METHODS

### *X. laevis* F-actin-intact extracts

F-actin-intact *X. laevis* extracts were prepared as described previously, with some modifications (Field et al., 2017; Good and Heald, 2018). Briefly, *X. laevis* eggs were washed with 1× Marc's Modified Ringer's (MMR) media (0.1 mM EDTA, 0.1 M NaCl, 2 mM KCl, 2 mM CaCl_2_, 1 mM MgCl_2_, 5 mM HEPES, pH 7.8) and dejellied with cysteine (2% w/v L-cysteine, pH 7.8) at 16°C. Dejellied eggs were washed with cytostatic factor extract buffer (CSF-XB; 100 mM KCl, 0.1 mM CaCl_2_, 1 mM MgCl_2_, 50 mM sucrose, 10 mM HEPES, 5 mM EGTA, pH 7.7), followed by washing with CSF-XB+, which is CSF-XB containing 1 µg/ml LPC [leupeptin (Sigma-Aldrich, EI8), pepstatin A (Sigma-Aldrich, EI10), chymostatin, (Sigma-Aldrich, EI6)]. The eggs were packed at 1200 ***g*** for 45 s and the excess buffer was aspirated. The eggs were then crushed at 15,000 ***g*** for 15 min at 16°C in a centrifuge containing a Beckman SW50.1 rotor. The straw-colored cytoplasmic layer was drawn out using an 18G syringe. The extract was supplemented with 1 µg/ml LPC and kept on ice. All *Xenopus* procedures and studies were conducted in compliance with the US Department of Health and Human Services Guide for the Care and Use of Laboratory Animals. Protocols were approved by the University of Wyoming Institutional Animal Care and Use Committee (Assurance #A-3216-01).

### Extract supplements

Recombinant GST–mCherry–NLS, GST–GFP–NLS, Lamin A and Lamin B3 were expressed and purified as previously described (Chen et al., 2019; Jevtić et al., 2015; Levy and Heald, 2010). LifeAct–GFP was used as an F-actin probe (Pelletier et al., 2020) and purified as previously described (Belin et al., 2015), with the only modification being that the protein was eluted in extract buffer (XB; 100 mM KCl, 0.1 mM CaCl_2_, 1 mM MgCl_2_, 50 mM sucrose, 10 mM HEPES, pH 7.8) for compatibility with egg extracts. The following working concentrations in extracts were used: 0.04–0.15 mg/ml GST–GFP–NLS, 0.04–0.15 mg/ml GST–mCherry–NLS, 2 µg/ml Hoechst 33342 (14533, Sigma-Aldrich), 280 nM Lamin A, 30–80 nM GFP–Lamin B3, 500–1000 nM LifeAct–GFP, 5–10 µg/ml Alexa Fluor 594-tagged mAb414 (682202, BioLegend) and 0.5 mg/ml rhodamine–actin (AR05-B, Cytoskeleton, Inc.).

### Nuclear assembly in F-actin-intact extracts and immunofluorescence

For every 100 µl of freshly prepared extract, we added 2 µl energy mix [190 mM creatine phosphate disodium (2380, Sigma-Aldrich), 25 mM ATP disodium salt (A2383, Sigma-Aldrich), 25 mM MgCl_2_], 1.5 µl cycloheximide stock solution [10 mg/ml cycloheximide (94271, Amresco) in XB], 1.5 µl Ca^2+^ stock (50 mM CaCl_2_, 0.5 M KCL, 5 mM MgCl_2_) and demembranated *Xenopus* sperm nuclei (1000/µl). Only extracts capable of nuclear formation and robust mitotic gelation-contraction were used for experiments (Field et al., 2017). *Xenopus* extract nuclei were imaged 90 min after setting up the nuclear assembly reaction unless otherwise stated. For live imaging, 10 µl of nuclear assembly reaction solution were supplemented with fluorescent probes as indicated under ‘Extract supplements’, pipetted on a clean glass slide, overlaid with a 22 mm×22 mm coverslip, sealed with VALAP (1:1:1, Vaseline, lanolin and paraffin wax) and imaged at room temperature. In some cases, nuclei were fixed and spun onto coverslips for immunofluorescence as described previously (Mishra and Levy, 2022). The following primary antibodies were used: anti-His-6× (1:500, Invitrogen, MA1-21315), anti-mouse mAb414 (1:1000, BioLegend, 902901) and anti-rabbit Nup53 (1:500, obtained from Richard Wozniak, University of Alberta, Edmonton, Canada; Hawryluk-Gara et al., 2005). The following secondary antibodies were used: anti-rabbit Alexa Fluor 568 (1:1000, Invitrogen, A11011), anti-mouse Alexa Fluor 568 (1:1000, Invitrogen A11031) and anti-rabbit Cy5 (1:500, Abcam, ab6564). F-actin was stained using 4 U/ml of Alexa Fluor 488 phalloidin (Invitrogen, A12379).

### siRNAs and plasmids

The following plasmids were used: control plasmid pEmCherry-C2 (a gift from Anne Schlaitz, University of Heidelberg, Heidelberg, Germany); nuclear-targeted actin plasmids pmCherry-C1 actin-3×NLS P2A mCherry (Addgene #58475, deposited by Dyche Mullins) and pmCherry-C1 R62D actin-3×NLS P2A mCherry (Addgene #58477, deposited by Dyche Mullins) (Belin et al., 2015); nuclear actin-chromobody-GFP plasmid (pnAC-TagGFP; Chromotek, acg-n); and pcDNA3.1-mCherry-Exp6 (gifted by Kei Miyamoto, Kindai University, Osaka, Japan; Okuno et al., 2020). Negative Control DsiRNA (siSCR; Integrated DNA Technologies, 51-01-14-04) was used as a negative control for Lamin A knockdowns. Lamin A knockdown was performed using predesigned Dicer-Substrate siRNA (siLMNA; Integrated DNA Technologies, hs.Ri.LMNA.13.3), with the following duplex sequences: 5′-rGrGrUrGrArGrGrCrCrArArGrArArGrCrArArCrUrUrCrAGG-3′ and 5′-rCrCrUrGrArArGrUrUrGrCrUrUrCrUrUrGrGrCrCrUrCrArCrCrUrA-3′.

### HeLa cell culture and transfection

HeLa cells were obtained from the American Type Culture Collection and H2B–GFP tagged HeLa cells were a gift from Jay Gatlin (University of Wyoming, WY). These cell lines were grown in Eagle's Minimal Essential Medium (MEM; 30-2003, ATCC) supplemented with 10% v/v fetal bovine serum (S11150H, Atlanta Biologicals) and 50 IU/ml penicillin/streptomycin at 37°C with 5% CO_2_. For co-transfection of plasmids and siRNA, Lipofectamine 3000 (Invitrogen, L3000001) was used according to the manufacturer's protocol. Cells were seeded at 2.1×10^5^ cells/well in a 6-well culture dish on acid-washed coverslips. After the cells reached 70–90% confluency, they were transfected with 1.25 µg plasmid (pEmCherry-C2, pmCherry-C1 actin-3×NLS P2A mCherry, pmCherry-C1 R62D actin-3×NLS P2A mCherry) and 37.5 µM siRNA (siSCR or siLMNA) per well. The media was replaced 24 h after transfection, and the coverslips were fixed 48 h after transfection and processed for immunofluorescence. For exportin-6 expression experiments, cells were seeded at 1.5×10^5^ cells/well, grown to 50–60% confluency and transfected with 37.5 µM siRNA (siSCR or siLMNA). After 24 h, cells were transfected with 2.5 µg pcDNA3.1-mCherry-Exp6 or pEmCherry-C2 as a control. After another 24 h, the coverslips were fixed and processed for immunofluorescence.

### Immunofluorescence and live imaging of HeLa cells

After 48 h of transfection, coverslips were washed three times with PBS for 5 min each, fixed with 4% paraformaldehyde for 15 min and washed three times again with PBS for 5 min each. Then the cells were permeabilized with 0.1% Triton X-100 in PBS (v/v) for 10 min and washed three times with PBS for 5 min each. For blocking, cells were incubated with 10% goat serum (Sigma-Aldrich, G9023) supplemented with 0.3 M glycine for 1 h at room temperature. The cells were then incubated overnight at 4°C with the primary antibodies diluted in 2% goat serum in PBS. The next day, cells were washed three times in PBS for 5 min each and then incubated with the secondary antibodies diluted in 2% goat serum in PBS and supplemented with 10 µg/ml Hoechst 33342 for 1 h at room temperature. Cells were again washed three times with PBS for 5 min each, followed by three brief washes with distilled water. Finally, the coverslips were mounted onto glass slides with Vectashield (Vector Laboratories H-1000), sealed with nail polish and stored at 4°C or imaged immediately. The following primary antibody was used: anti-Lamin B1 antibody (1:500, Abcam, ab16048). The following secondary antibodies were used: goat anti-rabbit immunoglobulin conjugated with Alexa Fluor 488 (1:1000, Invitrogen, A11008) or Cy5 (1:250, Abcam, ab6564).

For synchronization of HeLa cells for live imaging, cells were treated with 20 µM lovastatin (mevinolin, Sigma-Aldrich, M2147) for 24 h and then released from G1 arrest with 6 mM mevalonolactone (Sigma-Aldrich, M4667) for 24 h (Ma and Poon, 2017). Synchronized cells were transferred to µ-slide two-well glass-bottomed chambered coverslips (ibidi, 80287) and grown to 70–80% confluency. Cells were then transfected as indicated, the media was replaced after 24 h and live imaging was performed for 36 h (i.e. up to 60 h post transfection). Cell cycle phase analysis was based on the time elapsed after mitosis (Hahn et al., 2009).

### Microscopy and image analysis

Wide-field microscopy was performed using an Olympus BX63 upright widefield epifluorescence microscope with a high-resolution Hamamatsu ORCA-Flash 4.0 digital CMOS camera. Olympus objectives PLanApoN 2× (NA 0.08, air), UPLanFLN 20× (NA 0.5, air) and UPLanSApo 40× (NA 1.25, silicon oil) were used. The *xy* and *z* positions were controlled by a fully motorized Olympus stage. Confocal and super-resolution microscopy was performed using an Olympus IX83 (IXplore SpinSR) spinning disk super-resolution and confocal microscope (Yokogawa CSW-W1 SoRA) with a pinhole size of 50 µm and equipped with ORCA-Fusion Digital CMOS camera. The *xy* and *z* positions were controlled by a fully motorized Olympus stage. Olympus objectives UPLanXApo 20× (NA 0.8, air), UPLanSApo 40× (NA 1.25, silicon oil) and UPLanSApo 100× (NA 1.35, silicon oil) were used. Live imaging of HeLa cells was performed using an Olympus IX81 spinning disk confocal microscope with CSU-X1 confocal head with a pinhole size of 50 µm and a Hamamatsu ORCA-Flash 4.0 digital CMOS camera. Cells grown on glass-bottomed chambered coverslips were maintained at 37°C and 5% CO_2_ using a stage top incubator (Tokai Hit, INUBG2A-ZILCS). The Olympus objective UPLanSApo 20× (NA 0.75, air) was used. The *xy* and *z* positions were controlled by a fully motorized Olympus stage. Images were acquired using Olympus CellSens imaging software for widefield, confocal and super-resolution microscopy, and Metamorph software for live imaging.

For quantification, we generally used manual segmentation to define features of interest because automatic thresholding was at times inaccurate. All quantifications were performed using ImageJ/Fiji. For most HeLa cell experiments, nuclear circularity was measured for cells successfully transfected with mCherry, whereas for inhibitor treatments, images were randomly acquired across the coverslip and all nuclei in each image were quantified. Nuclear circularity for fixed HeLa cells was quantified by manual segmentation of Lamin B1-stained nuclei. For *Xenopus* extract experiments, nuclear circularity and cross-sectional areas were measured by manual segmentation of nuclei importing mCherry–NLS. For nuclear volume measurements, *z*-stack images for mCherry–NLS fluorescence were acquired with 1-µm *z*-slice thickness and the volumes of each nuclear slice were summed. For intensity measurements, images were acquired with the same exposure time, features of interest were manually segmented and background-subtracted fluorescence intensities were quantified. To measure total nuclear actin intensity, nuclei were manually outlined and the intensity within the whole nuclear area was measured. To measure actin, Lamin B3 and NPC intensity at the NE only, the nuclear rim was manually traced (excluding the nucleoplasmic signal) and the fluorescence signal intensity along the line was measured. To measure nucleoplasmic actin intensity, a box was drawn in the middle of the nucleus excluding the rim, and the intensity within the box was measured. For line scan measurements of F-actin and NPCs (Fig. 3), a straight line was drawn across the nucleus at regions where staining for both NPCs and F-actin was prominent and intensities were measured across the line for both channels. Generally, we normalized intensity values to controls to simplify data presentation.

### Inhibitor treatments

For inhibitor treatments in HeLa cells, 48 h after transfection with siLMNA, cells were incubated in media containing 25 µM SMIFH2 (Tocris, 4401) or 100 µM CK666 (Tocris, 3950) at 37°C for 1 h. Then, the inhibitor-containing media was replaced with fresh Eagle's MEM and the cells were fixed for immunofluorescence. Inhibitor concentrations were selected based on previous studies (Bovellan et al., 2014; Isogai et al., 2015). For *Xenopus* extract experiments, inhibitors were typically added to nuclear assembly reactions 45 min after initiation, as some inhibitors can affect NPC formation (Parisis et al., 2017). Nuclei were then imaged 45 min after inhibitor addition. For cytochalasin B, different concentrations of the inhibitor were added to the extract at the beginning of the reaction and nuclei were imaged 90 min after cytochalasin B addition. The working inhibitor concentrations were: 1–100 ng/µl cytochalasin B (Sigma-Aldrich, C6762), 500 µM SMIFH2 (Sigma-Aldrich, S4826), 500 µM CK-666 (Tocris, 3950), 100–200 µM CK-636 (MedChemExpress, HY-15892), 0.1–50 µM CK-869 (MedChemExpress, HY-16927), 100 µM blebbistatin (HY-13441), 100 µM ML-7 hydrochloride (MedChemExpress, HY-15417), 100 µM MLCK inhibitor peptide 18 (MedChemExpress, HY-P1029). For SMIFH2, only stock solutions less than 1 month old were used, as the inhibitor starts to lose its potency after a month. However, for other inhibitors, the manufacturers' recommendations for stock stability were followed. In general, higher inhibitor concentrations are required in *Xenopus* extract compared to those required in intact cells, as several hundred micromolar of inhibitors are often needed to cause effective inhibition in *Xenopus* extract. This might be because inhibitors are rendered ineffective in extracts when absorbed into abundant membrane compartments and/or because active transport in cells results in higher intracellular concentrations of inhibitors (Parisis et al., 2017).

### Western blots

HeLa cell lysates were prepared 48 h post transfection. The cells were washed twice with PBS, then suspended in RIPA buffer [50 mM Tris-HCl pH 7.5, 150 mM NaCl, 1% NP-40, 0.5% sodium deoxycholate, 0.1% SDS, 1× protease inhibitor cocktail (Sigma-Aldrich, S8830), 1 mM dithiothreitol]. Then, the cells were collected using a cell scraper into 1 ml tubes and lysed on ice for 30 min. Afterwards, the cell lysate was centrifuged at 16,000 ***g*** for 10 min at 4°C and the supernatant was collected. The protein concentration of the cell lysate was measured using the EZQ Protein Quantitation Kit (Invitrogen, R33200). Then, 8 µg of protein lysate was mixed with SDS-PAGE loading buffer (0.05% Bromophenol Blue, 0.1 M dithiothreitol, 10% glycerol, 2% SDS, 0.05 M Tris-HCl, pH 6.8) and boiled for 10 min. Proteins were separated on 10% Mini-PROTEAN TGX Precast Gels (Bio-Rad) and transferred to Immobilon-FL polyvinylidene difluoride membranes (Merck Millipore, IPFL00010) using a semi-dry transfer apparatus. Blocking was performed with Odyssey Blocking Buffer (LI-COR Biosciences, 927-40000) for 1 h at room temperature, followed by overnight incubation at 4°C with the primary antibody diluted in a 2:1 solution of PBS containing 0.1% Tween 20 (PBST) and Odyssey Blocking Buffer. The membrane was washed three times with PBST for 5 min each and incubated 1 h at room temperature with secondary antibodies diluted in Odyssey Blocking Buffer containing 0.01% SDS and 0.1% Tween 20. The membrane was again washed three times with PBST for 5 min each. After a final PBS rinse, the membrane was imaged using an Odyssey CLx instrument (LI-COR Biosciences). After imaging, the membrane was stained with Ponceau S to estimate the total protein per lane for normalization. Band intensities were quantified using ImageJ/Fiji. The following primary antibodies were used: anti-Lamin A/C antibody (1:1000, Santa Cruz Biotechnology, sc-376248) and anti-GAPDH antibody (1:10,000, Abcam, ab9485). The following secondary antibodies were used: IRDye 800CW anti-rabbit (1:20,000, LI-COR Biosciences, 926-32211) and IRDye 680RD anti-mouse (1:20,000, LI-COR Biosciences 925-68070).

### Statistical analyses

All raw data are presented in Table S1. Statistical analyses were performed for all experiments using GraphPad Prism and Anderson–Darling tests were used to assess whether data fit a normal distribution. For comparing two sets of data, two-tailed unpaired Student's *t*-tests (parametric) or Mann–Whitney tests (nonparametric) were performed. For comparing three or more sets of data, one-way ANOVA (parametric) with Tukey's post hoc test or Kruskal–Wallis tests (nonparametric) were performed. Grubbs' or Rout's tests were performed for outlier removal where indicated.

## Supplementary Material

10.1242/joces.259692_sup1 Supplementary information
