# Supplementary material for: Nuclear F-actin and Lamin A antagonistically modulate nuclear shape
Source: J Cell Sci. 2022 Jul 4;135(13):jcs259692. doi: 10.1242/jcs.259692 (PMC9377710; doi:10.1242/jcs.259692)
Supplement: Supplementary information [file joces-135-259692-s1.pdf]

Supplemental Figure 1

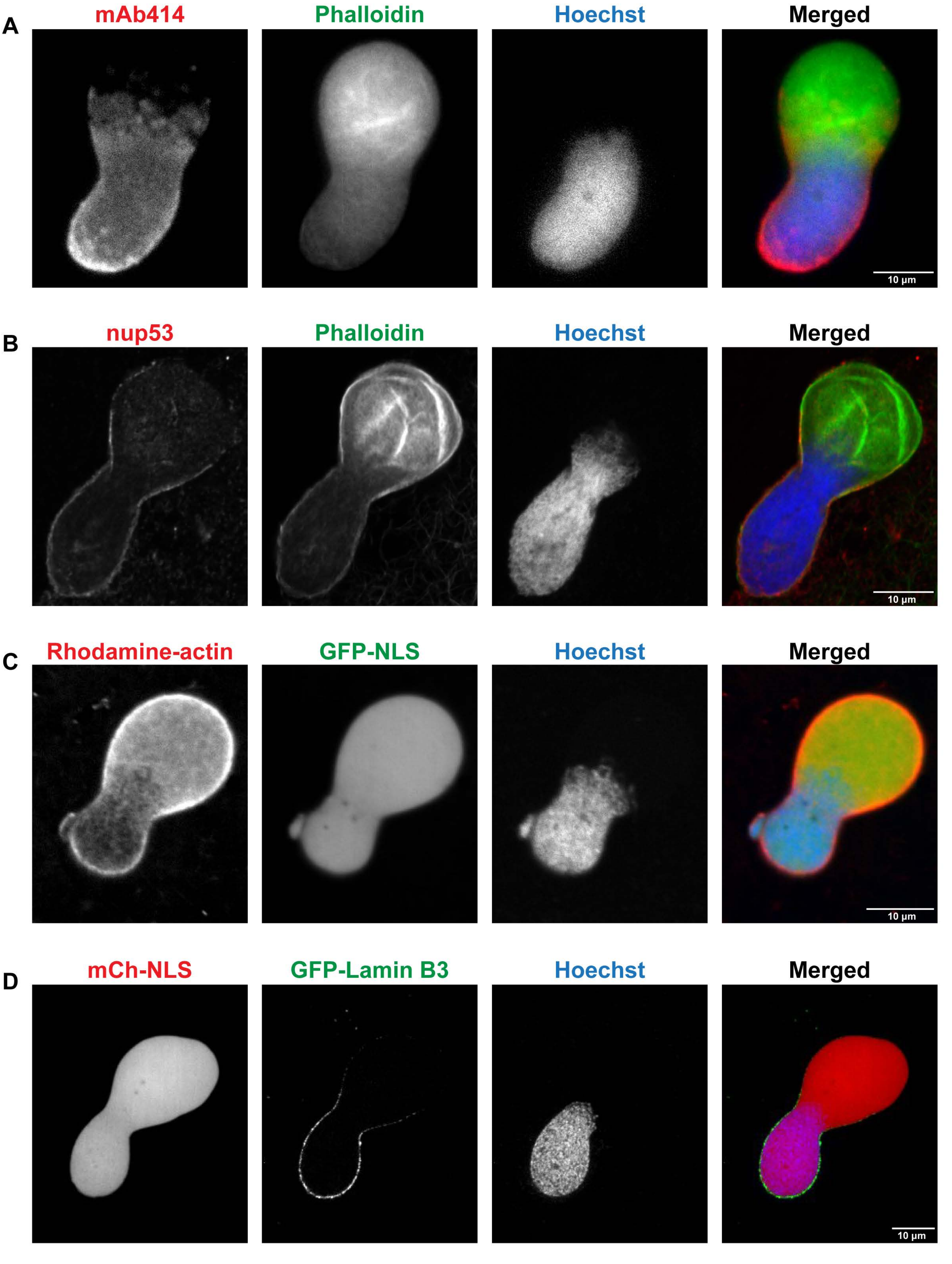

Fig. S1. Nuclear F-actin results in bilobed nuclear shape in both fixed and live nuclei in the absence of LifeAct-GFP. (A) For the experiments described in Figure 1 for F-actin intact extracts, nuclei were fixed, spun onto coverslips, and stained with mAb414 (red), Phalloidin (green), and Hoechst (blue). Representative widefield microscopy images are shown. **(B)** Nuclei were fixed as in (A) and stained with an anti-nup53 antibody (red), Phalloidin (green), and Hoechst (blue). Representative confocal microscopy images are shown. **(C)** Experiments were performed as in Figure 1 except that extract was supplemented with Rhodamine-actin (red), GFP-NLS (green), and Hoechst (blue). Representative live confocal microscopy images are shown. **(D)** Experiments were performed as in Figure 1 except that extract was supplemented with mCherry-NLS (red), GFP-Lamin B3 (green), and Hoechst (blue). Representative live confocal microscopy images are shown.

Supplemental Figure 2

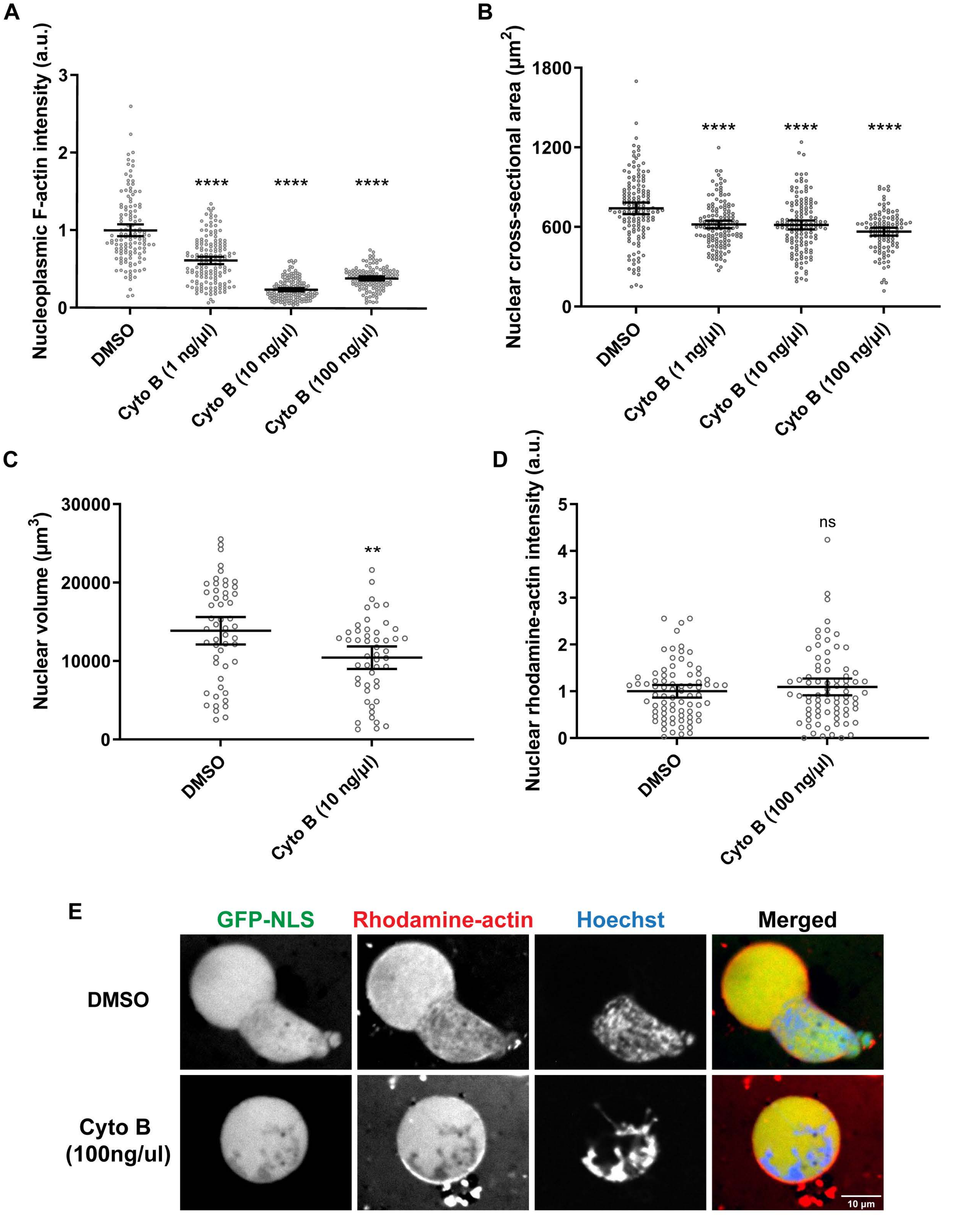

Journal of Cell Science • Supplementary information

Fig. S2. Nuclear F-actin promotes increased nuclear size. (A) For the experiments described in Figure 1, nucleoplasmic F-actin intensity was measured based on LifeAct-GFP signal within the nucleoplasm (excluding rim signal), normalized to DMSO controls. Images were acquired by widefield microscopy. Based on 2 independent experiments, the number of nuclei quantified was 129 DMSO nuclei, 149 Cyto B (1 ng/µl) nuclei, 164 Cyto B (10 ng/µl) nuclei, and 123 Cyto B (100 ng/µl) nuclei. **(B)** For the experiments described in Figure 1, nuclear cross-sectional area was measured based on mCherry-NLS signal from widefield microscopy images. Based on 2 independent experiments, the number of nuclei quantified was 141 DMSO nuclei, 133 Cyto B (1 ng/µl) nuclei, 141 Cyto B (10 ng/µl) nuclei, and 108 Cyto B (100 ng/µl) nuclei. **(C)** For the experiments described in Figure 1, confocal z-stack images were acquired based on mCherry-NLS signal and nuclear volume was quantified for 52 DMSO control nuclei and 50 nuclei from the 10 ng/µl cytochalasin treatment condition. **(D-E)** Experiments were performed as in Figure 1 except that extract was supplemented with Rhodamine-actin to visualize total actin (red), GFP-NLS (green), and Hoechst (blue). **(D)** Mean nuclear Rhodamine-actin signal intensity was measured for 77 nuclei for each condition and normalized to the DMSO control. **(E)** Representative images are shown. Mean values and 95% confidence interval (CI) error bars are shown. Student's t-tests were performed for (C) and (D). Nonparametric Kruskall-Wallis tests were performed for (A) and one-way ANOVA was performed for (B). Statistical significance is shown relative to DMSO controls. $P>0.05$ ns, $P\leq0.01$ **, $P\leq0.0001$ ****.

Journal of Cell Science • Supplementary information

Supplemental Figure 3

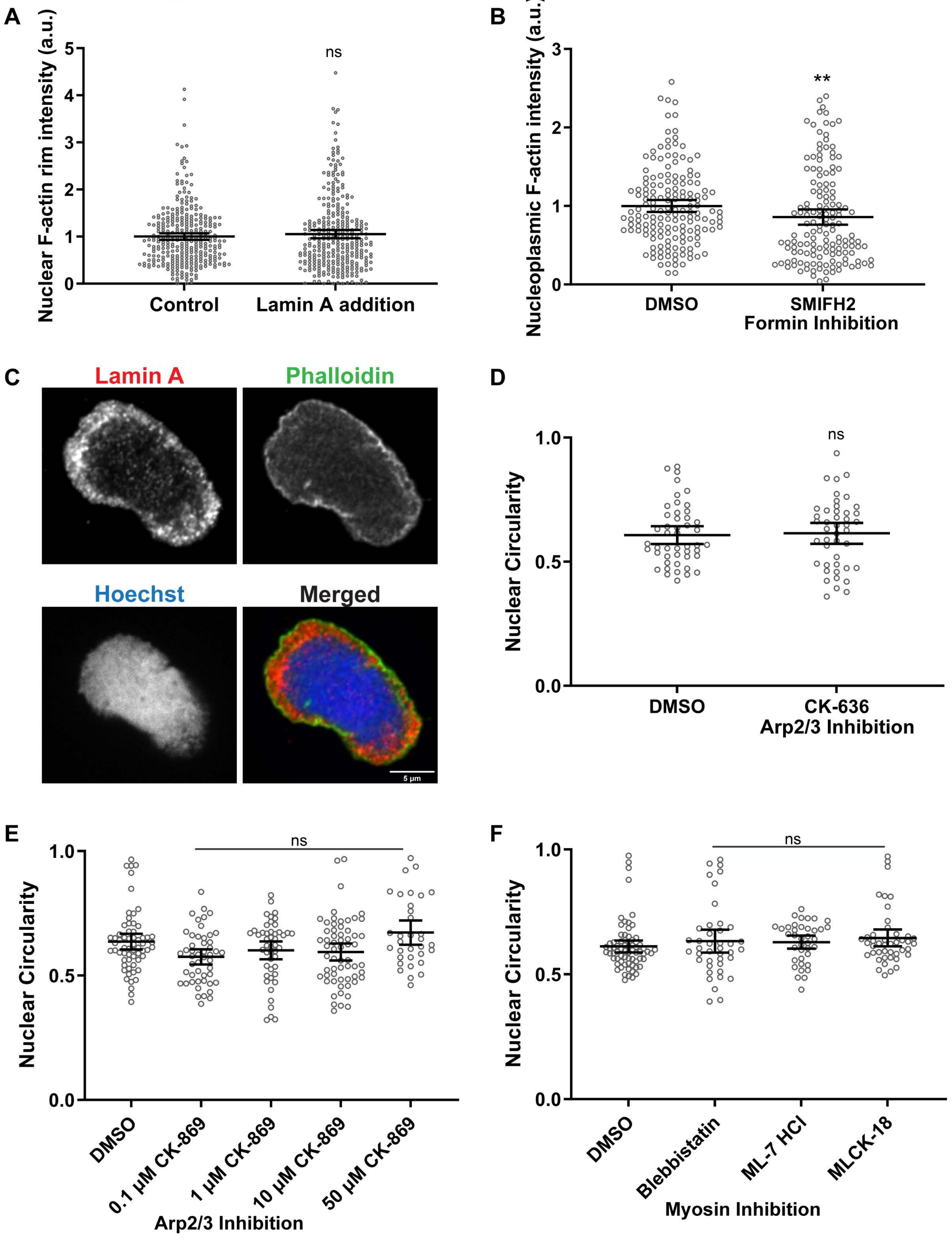

Fig. S3. Lamin A localization and inhibition of actin regulators in F-actin intact *Xenopus* egg extracts. (A) Experiments were performed as in Figure 4. F-actin at the nuclear rim was quantified based on LifeAct-GFP signal intensity from widefield microscopy images. Data are from 4 independent experiments with 320 nuclei for control and 322 nuclei for Lamin A addition. **(B)** Experiments were performed as in Figure 6. Nucleoplasmic F-actin intensity was measured based on LifeAct-GFP signal within the nucleoplasm (excluding rim signal) from widefield microscopy images. Based on 3 independent experiments, 160 DMSO nuclei and 145 SMIFH2 nuclei were quantified. **(C)** Experiments were performed as in Figure 4. For extracts supplemented with recombinant Lamin A, nuclei were fixed, spun down onto coverslips, and stained for His-tagged Lamin A (red), Phalloidin (green), and Hoechst (blue). Representative confocal microscopy images are shown. **(D)** Experiments were performed as in Figure 1 with F-actin intact extract except that 200 μM of Arp2/3 inhibitor CK-636 was added after 45 minutes of nuclear assembly. Nuclear circularity was measured for 47 DMSO nuclei and 44 CK-636 nuclei. **(E)** Experiments were performed as in Figure 1 with F-actin intact extract except that the indicated concentrations of Arp2/3 inhibitor CK-869 were added after 45 minutes of nuclear assembly. Nuclear circularity was measured for 62 DMSO nuclei, 49 CK-869 (0.1 μM) nuclei, 47 CK-869 (1 μM) nuclei, 61 CK-869 (10 μM) nuclei, and 33 CK-869 (50 μM) nuclei. **(F)** Experiments were performed as in Figure 1 with F-actin intact extract except that 100 μM of the indicated myosin inhibitors was added after 45 minutes of nuclear assembly. Nuclear circularity was measured for 69 DMSO nuclei, 41 Blebbistatin nuclei, 38 ML-7 HCl nuclei, and 44 MLCK-18 nuclei.

Mean values and 95% CI error bars are shown. Nonparametric Mann-Whitney tests were performed in (A), (B), and (D). Nonparametric Kruskall-Wallis tests were performed in (E) and (F). Statistical significance is shown relative to DMSO controls. P>0.05 ns, P≤0.01 **.

Journal of Cell Science • Supplementary information

Supplemental Figure 4

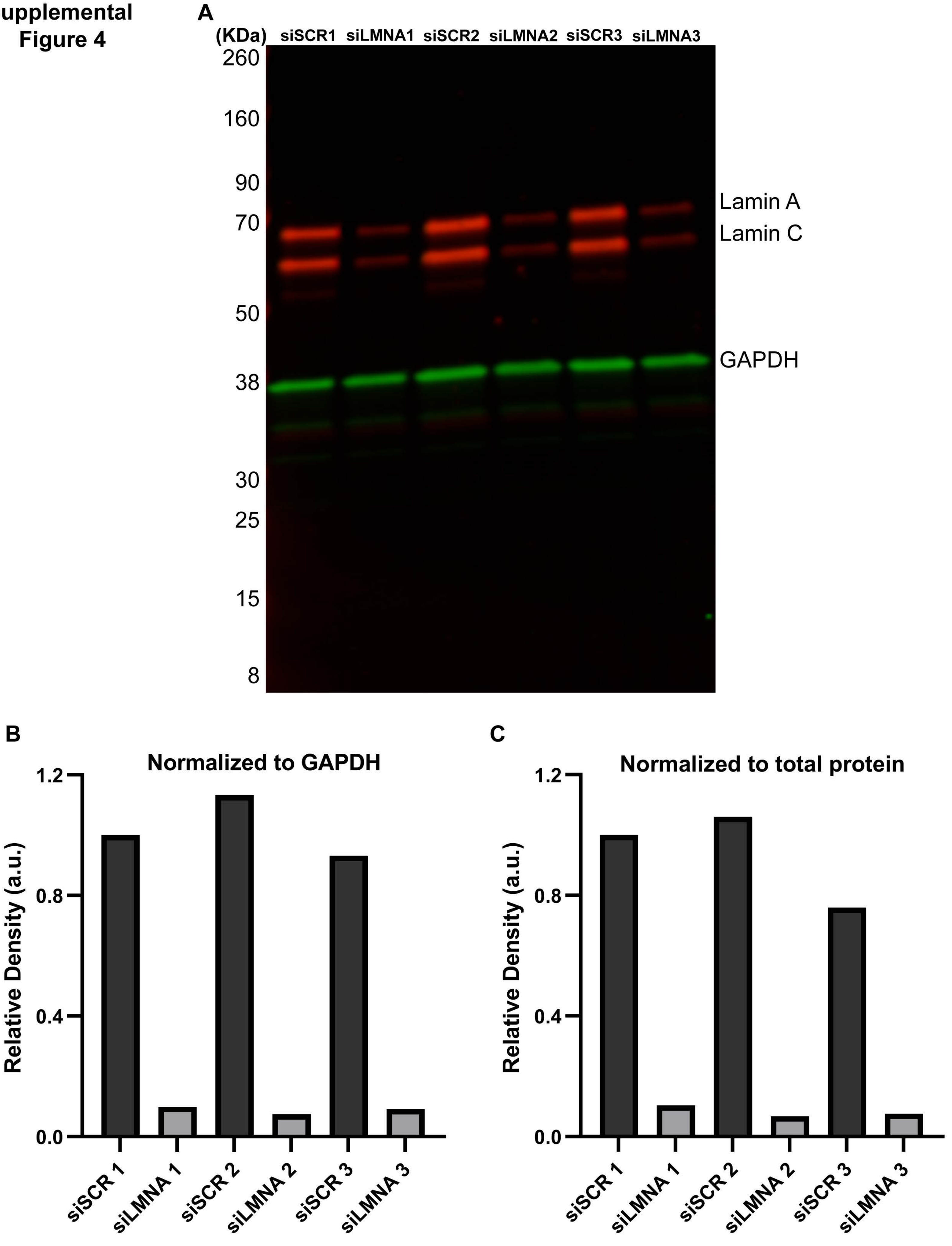

Journal of Cell Science • Supplementary information

Fig. S4. Confirmation of Lamin A/C knockdown in HeLa cells. (A) HeLa cells were transfected with a scrambled control (siSCR) or siRNA targeted against Lamin A/C (siLMNA). Cell lysates were subjected to western blot analysis, probing for Lamin A/C and GAPDH as a loading control. The numbers refer to three different replicates. **(B)** Lamin A/C band intensity was normalized to GAPDH band intensity for each sample. **(C)** Blots were stained with Ponceau to detect total protein. Lamin A/C band intensity was normalized to total protein band intensity for each sample.

Supplemental Figure 5

A

Extremely aberrant and multilobed nuclei (circularity <0.6) observed in ~16% of siLMNA+actinWT-NLS cells

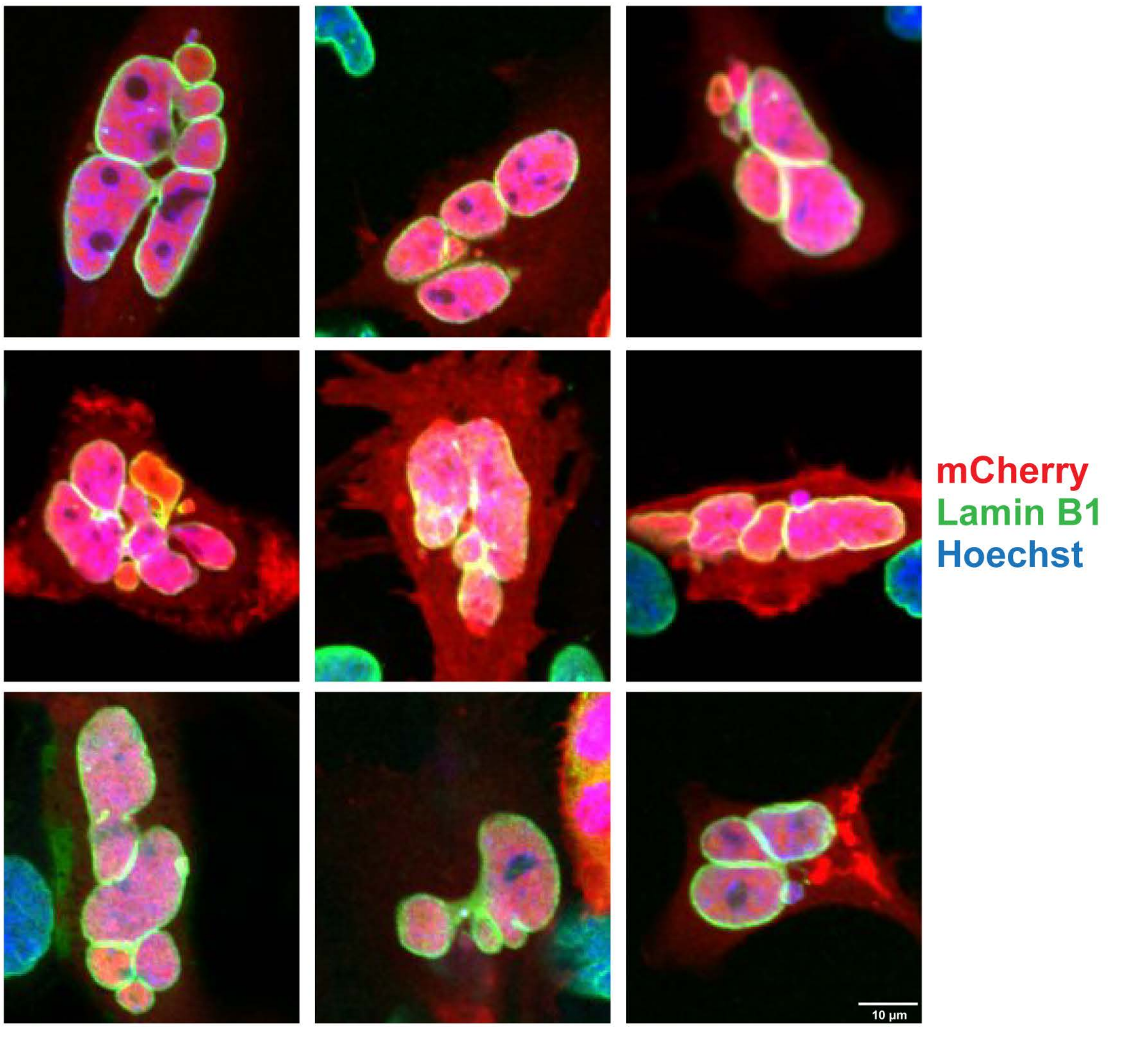

B

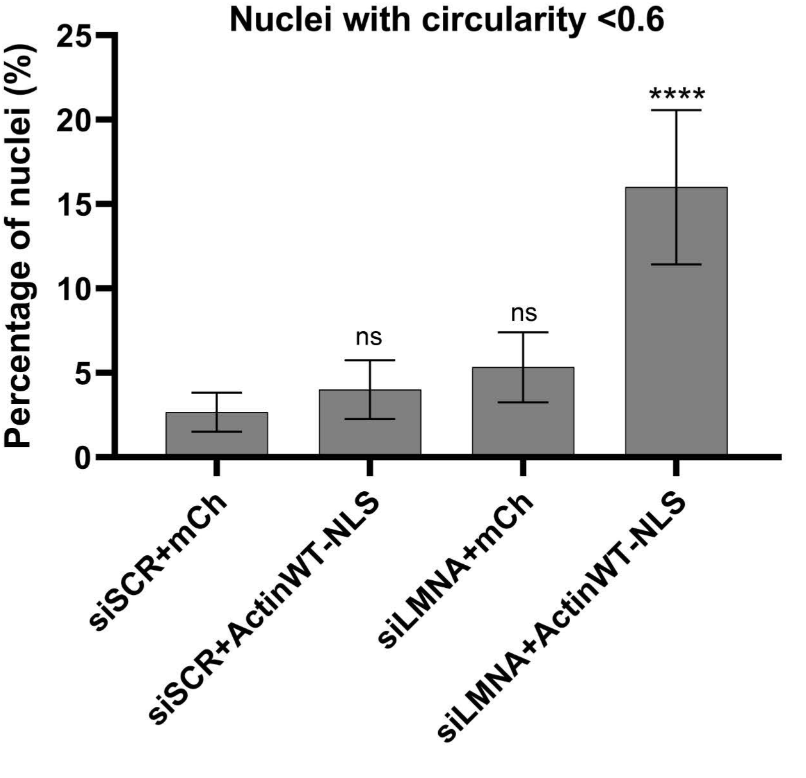

Journal of Cell Science • Supplementary information

Fig. S5. Extreme nuclear morphology phenotypes in HeLa cells. (A) Experiments were performed as described in Figure 5A. In ~16% of transfected Lamin A knockdown cells expressing nuclear-targeted actin, extremely aberrant or multilobed nuclei were observed (circularity below 0.6). **(B)** Based on the data presented in Fig. 5A-B, the percentage of extremely aberrant or multilobed nuclei (circularity below 0.6) was calculated for each condition based on 3 independent experiments. Mean values and SD error bars are shown: siSCR+mCh (2.7%±1.2%), siSCR+actinWT-NLS (4.0%±1.7%), siLMNA+mCh (5.3%±2.1%), and siLMNA+actinWT-NLS (16.0%±4.6%). Statistical significance relative to siSCR+mCh was assessed by contingency Chi-square tests. $P>0.05$ ns, $P\leq0.0001$ ****.

Journal of Cell Science • Supplementary information

Supplemental Figure 6

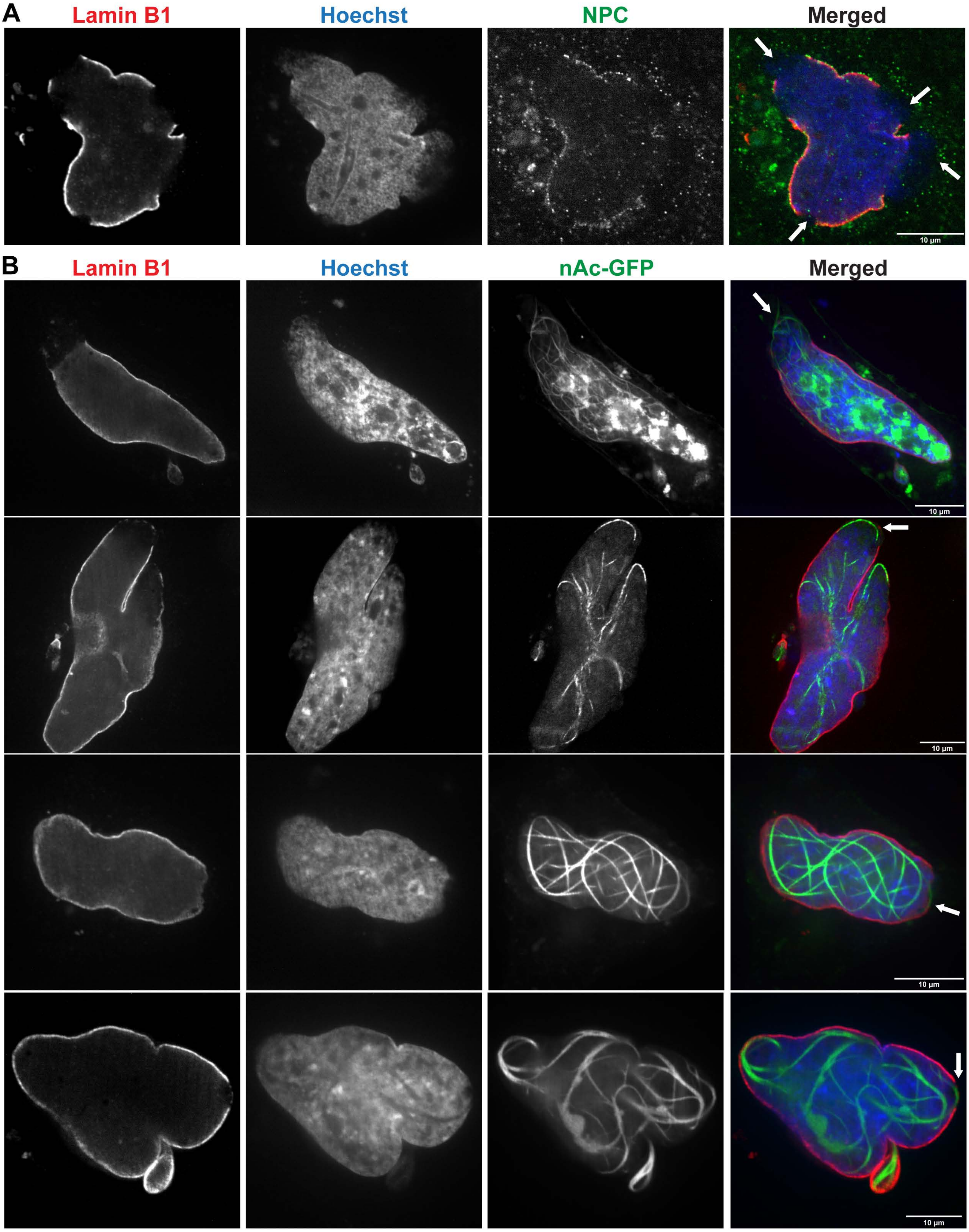

Journal of Cell Science • Supplementary information

Fig. S6. Differential distribution of NPCs, Lamin B1, DNA, and F-actin in Lamin A knockdown HeLa cells expressing nuclear-targeted actin. (A) Experiments were performed as described in Figure 5A for the siLMNA+actinWT-NLS condition. Fixed nuclei were stained for Lamin B1 (red), DNA (blue), and NPCs (green, mAb414) and imaged by SoRa super-resolution confocal microscopy. A representative nucleus with nuclear circularity below 0.6 is shown, where coinciding regions of low Lamin B1, NPC, and DNA intensity are evident (white arrows). This phenotype was observed in 59.7%±7.1% (mean±SD) of nuclei with nuclear circularity below 0.6, based on 3 independent experiments and analysis of 51 nuclei. **(B)** Experiments were performed as described in Figure 5A for the siLMNA+actinWT-NLS condition and including co-transfection with the nuclear actin marker nAC-GFP (green). Fixed nuclei were stained for Lamin B1 (red) and DNA (blue) and imaged by SoRa super-resolution confocal microscopy. Representative images are shown, and 114 nuclei from 3 independent experiments were analyzed. 35.5%±3.1% of transfected cells exhibited some accumulation of actin at the nuclear rim. Of those nuclei, 69.6%±2.5% exhibited regions of the NE where actin accumulation coincided with reduced amounts of Lamin B1 and DNA (white arrows).

Supplemental Figure 7

A

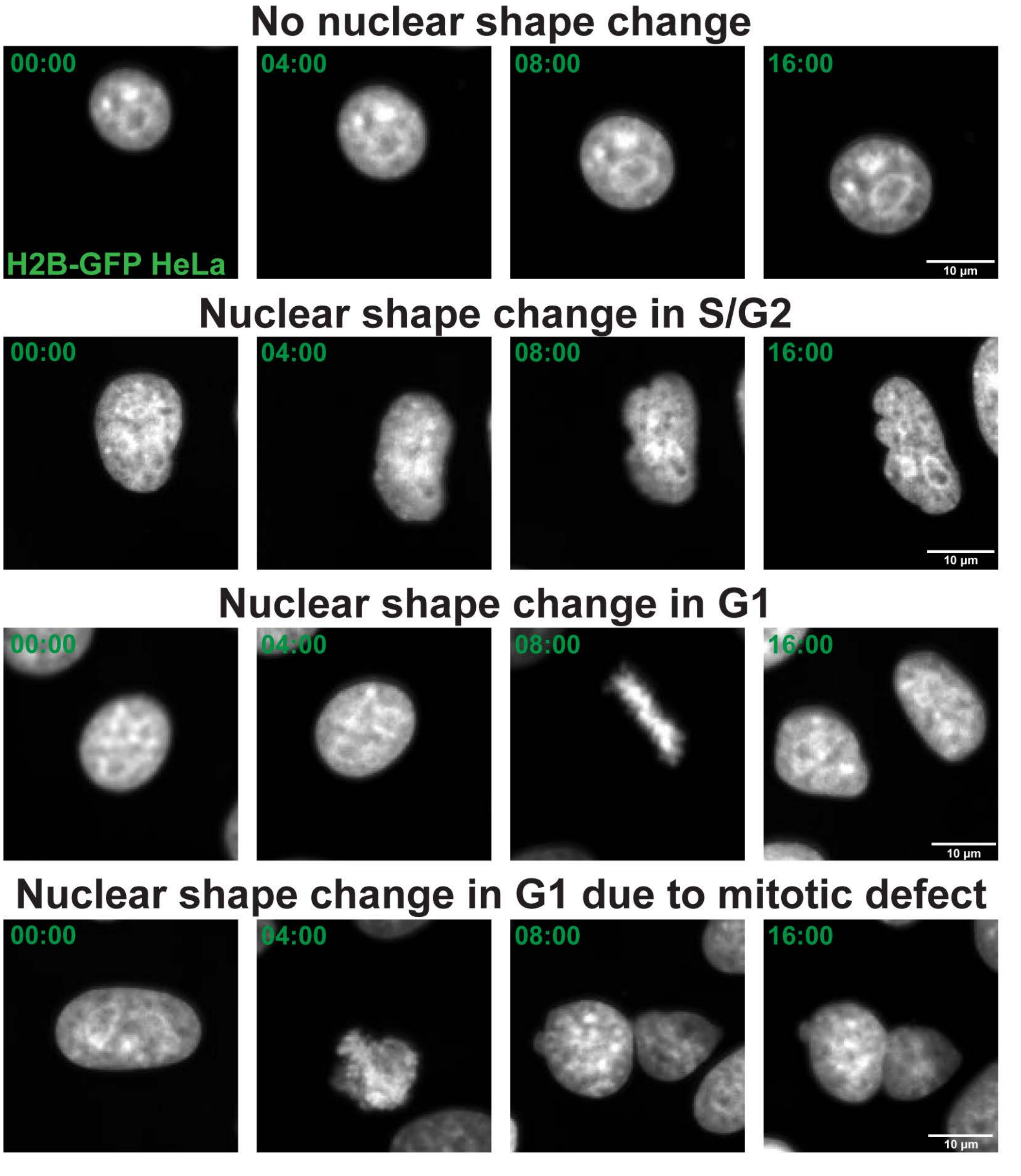

B

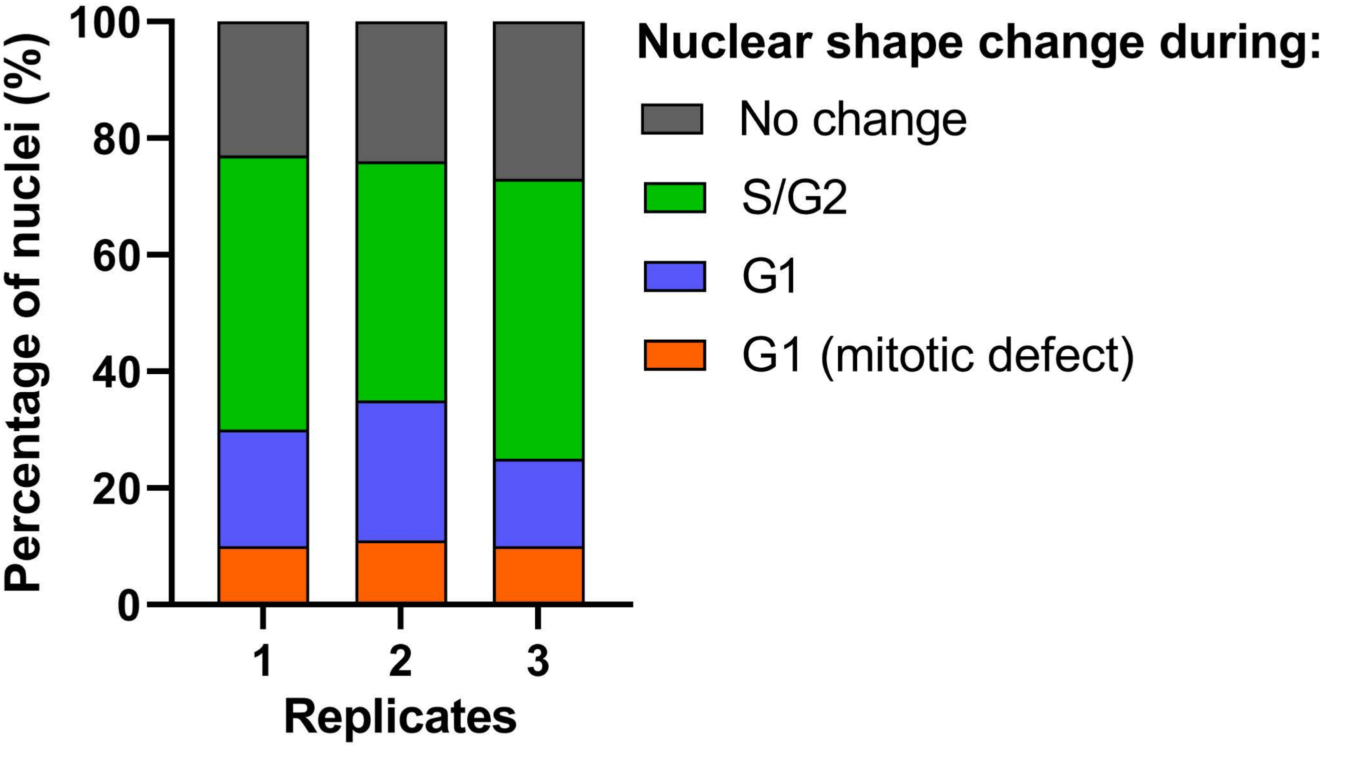

Journal of Cell Science • Supplementary information

Fig. S7. Time-lapse imaging of H2B-GFP tagged HeLa cells. G1-synchronized H2B-GFP HeLa cells were seeded on glass-bottom dishes, co-transfected with siRNA against Lamin A and the actinWT-NLS expression plasmid, and imaged live by confocal microscopy. Cell cycle stages were estimated based on time elapsed after mitosis (Hahn et al., 2009) . Nuclei were scored as undergoing a shape change if there was any deviation from the typical ovoid shape of HeLa cell nuclei, including nuclear elongation and lobulation. Cells were scored as having mitotic defects if metaphase plate formation was abnormal (e.g. Y-shaped metaphase plate), if mitosis was delayed (i.e. cells were arrested in metaphase for longer than 4 hours), and/or if obvious chromosome segregation defects were observed (e.g. lagging chromosomes, micronuclei, daughter nuclei that did not separate or were unequal in size). **(A)** Images from representative time-lapses are shown. Relative times are displayed as hours:minutes, starting at 00:00 in each case. Cropping at each time point was adjusted to account for cell migration. Nuclear shape change in S/G2 refers to nuclei that were round immediately after mitosis and subsequently underwent shape changes later in interphase. Nuclear shape change in G1 refers to nuclei that exhibited aberrant shapes immediately after mitosis in the absence of any obvious mitotic defects. (**B**) Based on 3 independent experiments, nuclei were categorized as shown in (A) for 300 nuclei. Mean±SD: 10.0%±0.4% for G1 (mitotic defect), 19.9%±4.8% for G1, 45.4%±4.4% for S/G2, and 24.7%±1.9% for No change.

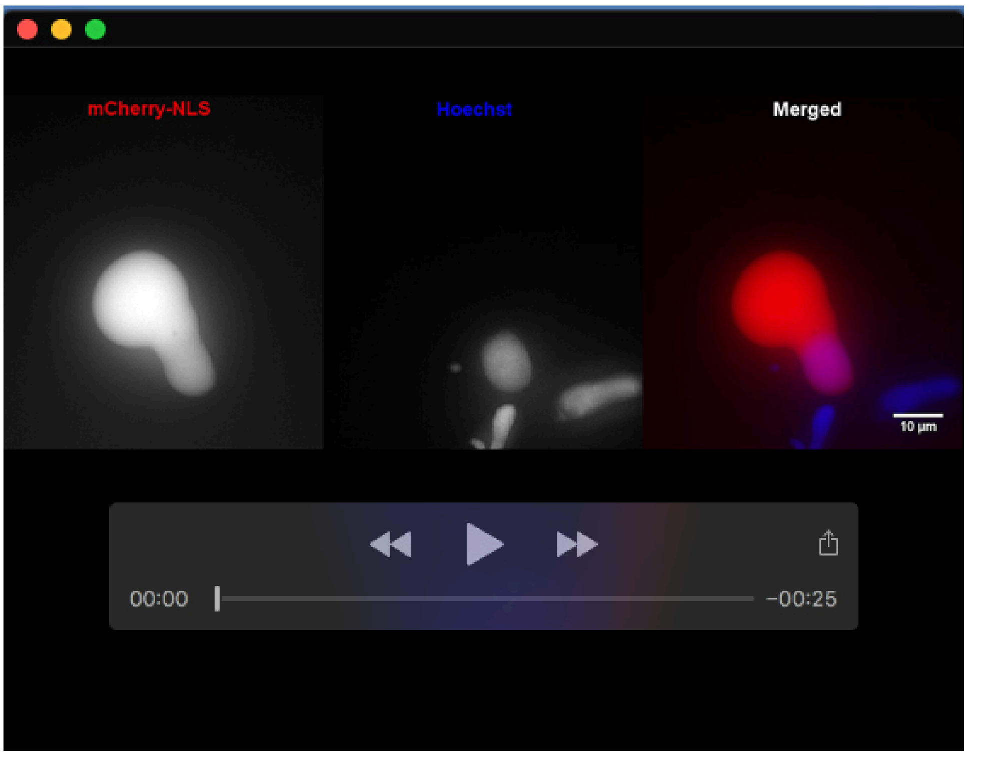

Movie 1. Formation of nuclear bilobes in F-actin intact *Xenopus* egg extracts. Nuclei were allowed to form in F-actin intact extract for 45 minutes prior to addition of mCherry-NLS (red) and Hoechst (blue). A 2-hour timelapse was acquired, imaging every two minutes.

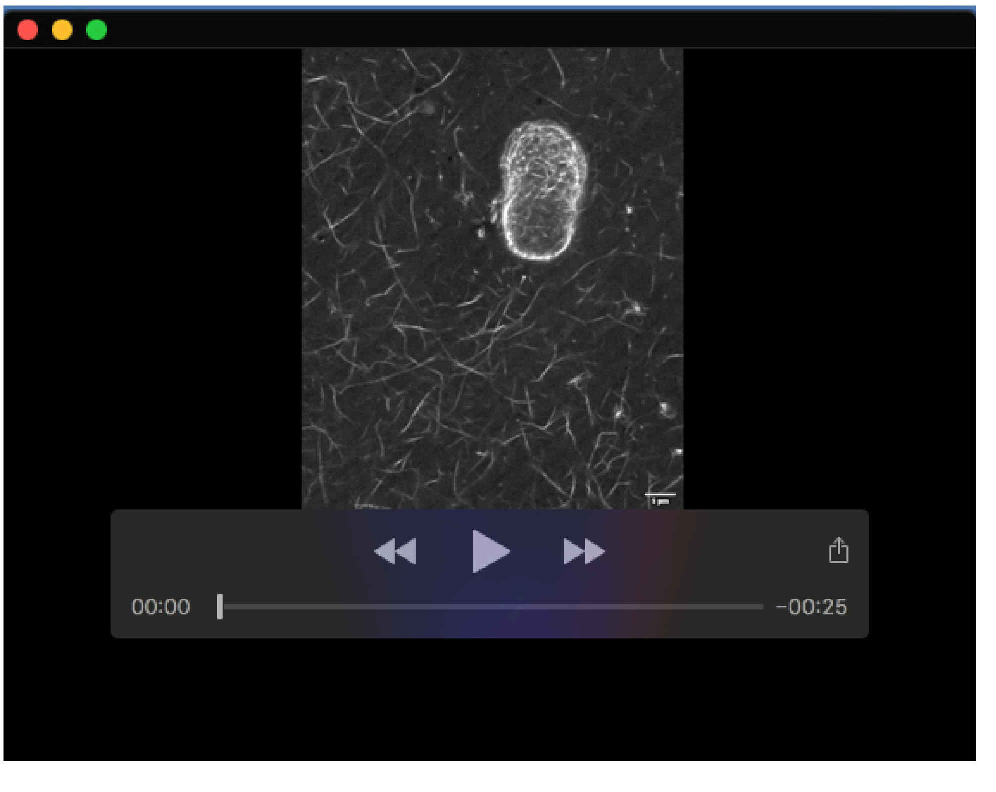

Movie 2. Nuclear F-actin dynamics in *Xenopus* egg extracts. LifeAct-GFP was added to actin-intact *X. laevis* egg extract just before nuclear assembly and a 30-minute timelapse was acquired, imaging every minute.

Journal of Cell Science • Supplementary information

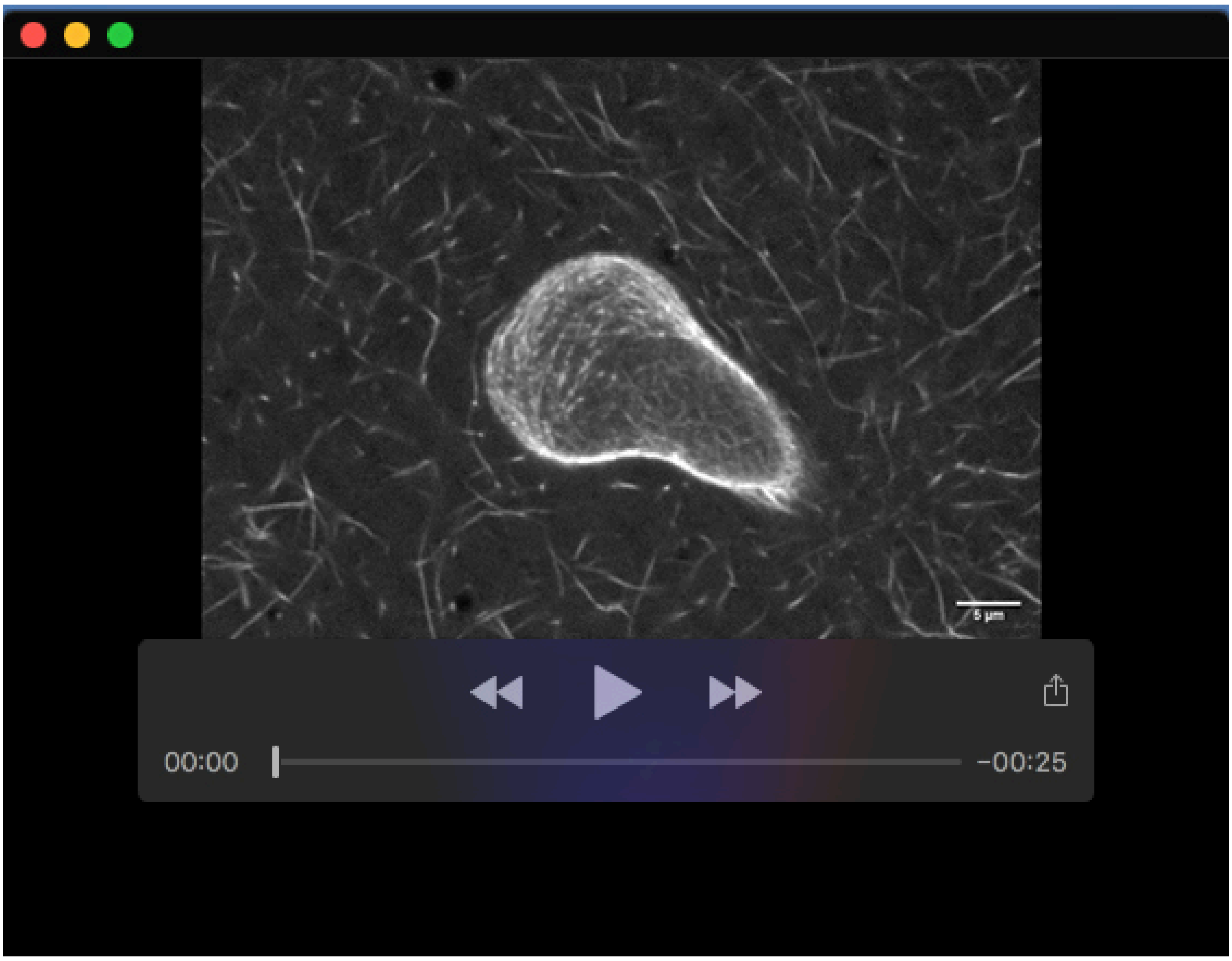

Movie 3. Nuclear F-actin dynamics in *Xenopus* egg extracts. LifeAct-GFP was added to actin-intact *X. laevis* egg extract just before nuclear assembly and a 26-minute timelapse was acquired, imaging every minute.

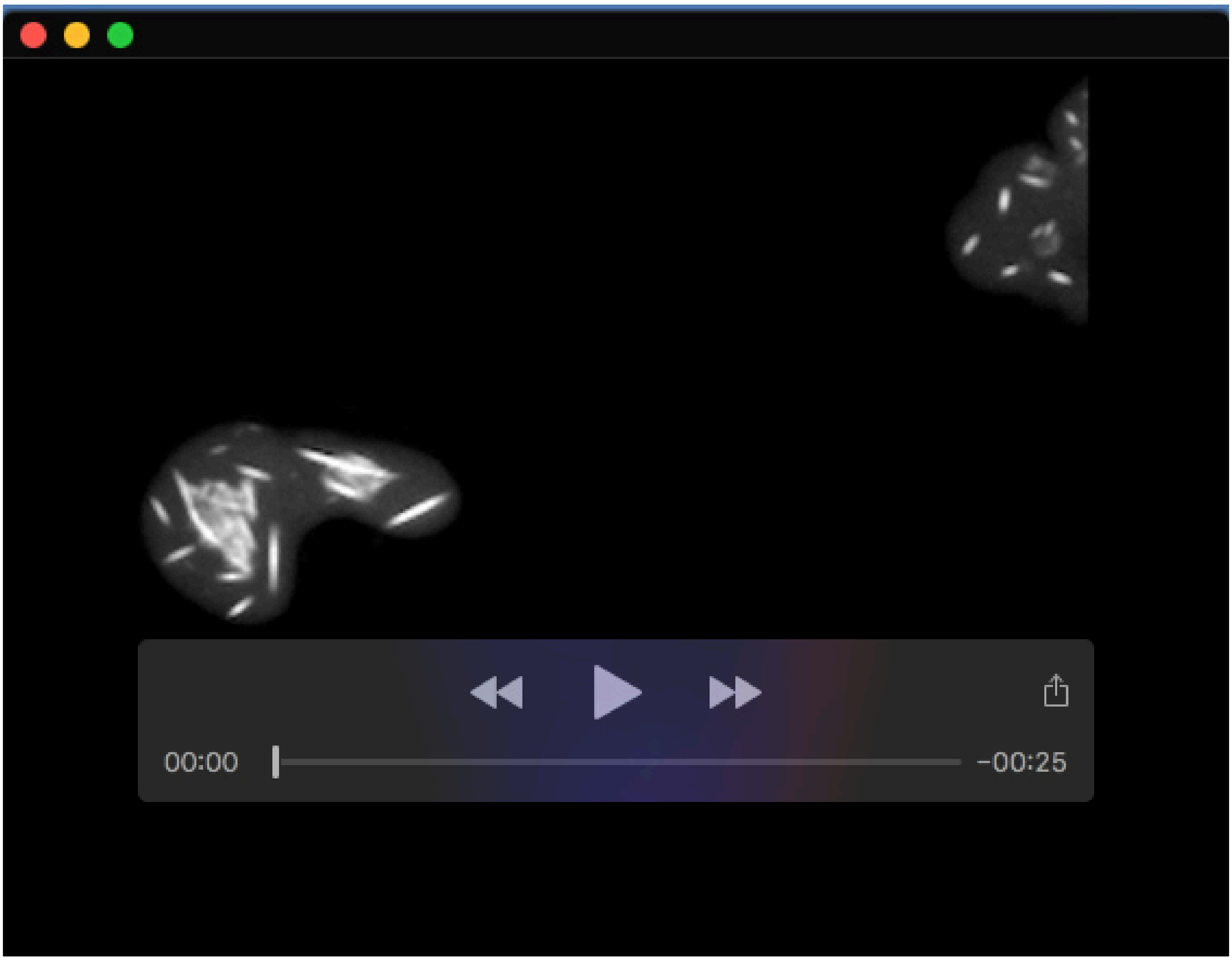

Movie 4. Nuclear F-actin dynamics in Lamin A knockdown HeLa cells expressing actinWT-NLS and nAC-GFP. G1-synchronized HeLa cells were seeded on glass-bottom dishes, co-transfected with siRNA against Lamin A and expression plasmids for actinWT-NLS and nAC-GFP, and imaged live by confocal microscopy 24 hours after transfection. A 36-hour timelapse was acquired, imaging every 4 hours. Nuclei were cropped at each time point to account for cell migration. The yellow arrowhead in the last frame indicates a site of actin accumulation at the nuclear rim, and frame 3 corresponds to mitosis.

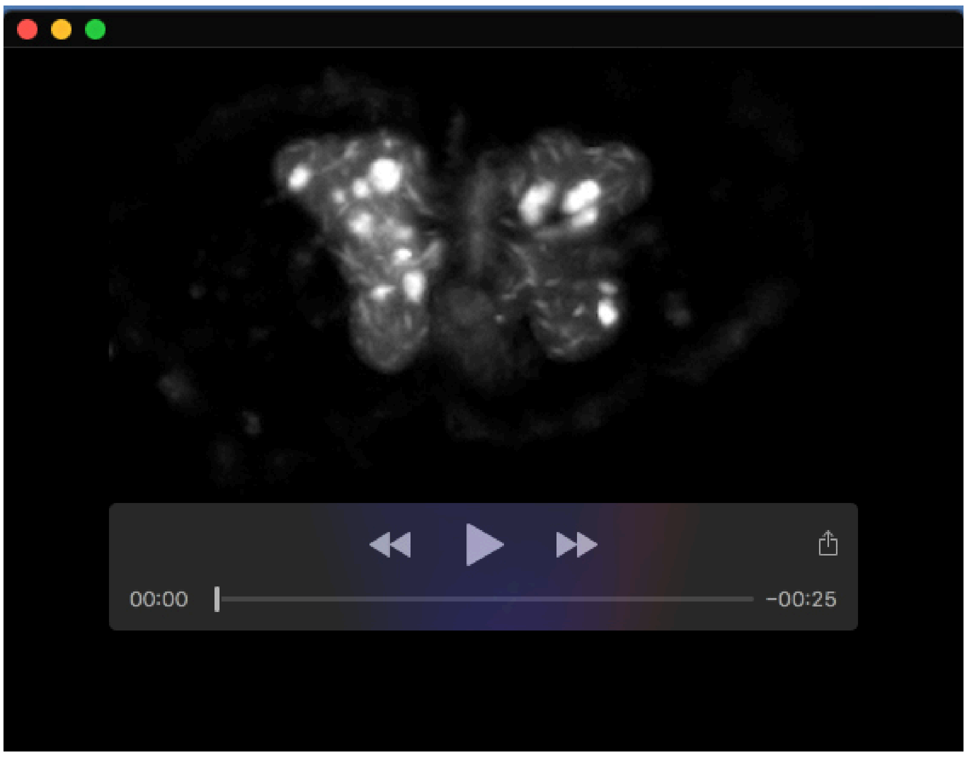

Movie 5. Nuclear F-actin dynamics in Lamin A knockdown HeLa cells expressing actinWT-NLS and nAC-GFP. G1-synchronized HeLa cells were seeded on glass-bottom dishes, co-transfected with siRNA against Lamin A and expression plasmids for actinWT-NLS and nAC-GFP, and imaged live by confocal microscopy 24 hours after transfection. A 20-hour timelapse was acquired, imaging every 4 hours. Nuclei were cropped at each time point to account for cell migration. The yellow arrowhead in the first frame indicates a site of actin accumulation at the nuclear rim, and frame 3 corresponds to mitosis.

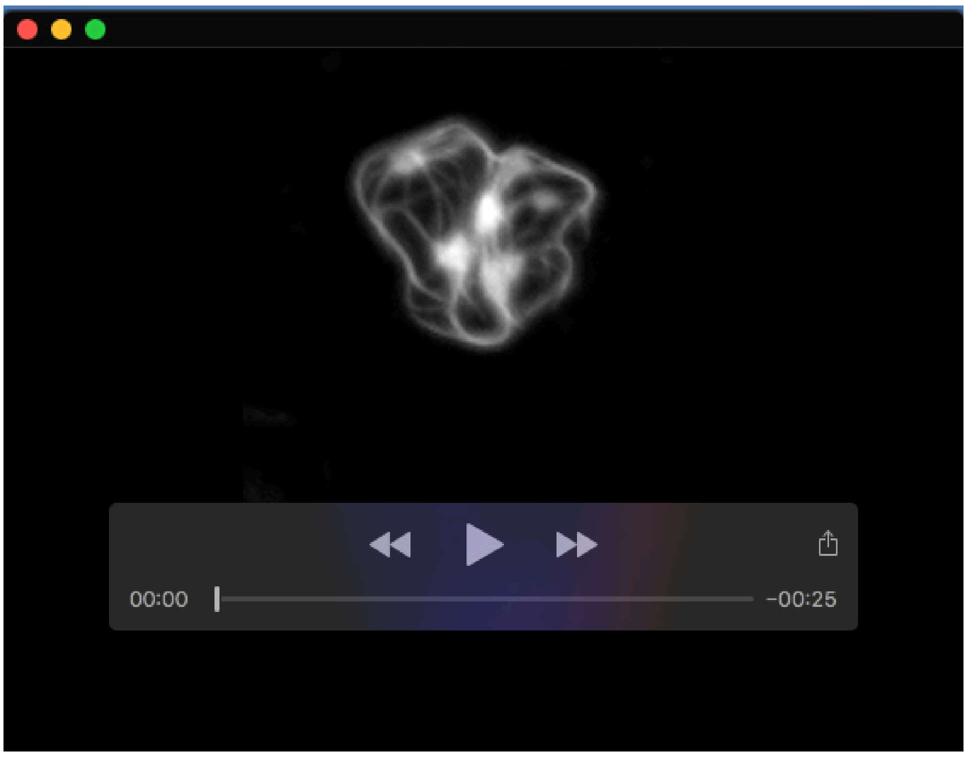

Movie 6. Nuclear F-actin dynamics in Lamin A knockdown HeLa cells expressing actinWT-NLS and nAC-GFP. G1-synchronized HeLa cells were seeded on glass-bottom dishes, co-transfected with siRNA against Lamin A and expression plasmids for actinWT-NLS and nAC-GFP, and imaged live by confocal microscopy 20 hours after transfection. A 2-hour timelapse was acquired, imaging every 10 minutes. The yellow arrowhead in the first frame indicates a site of actin accumulation at the nuclear rim, and only interphase is shown.

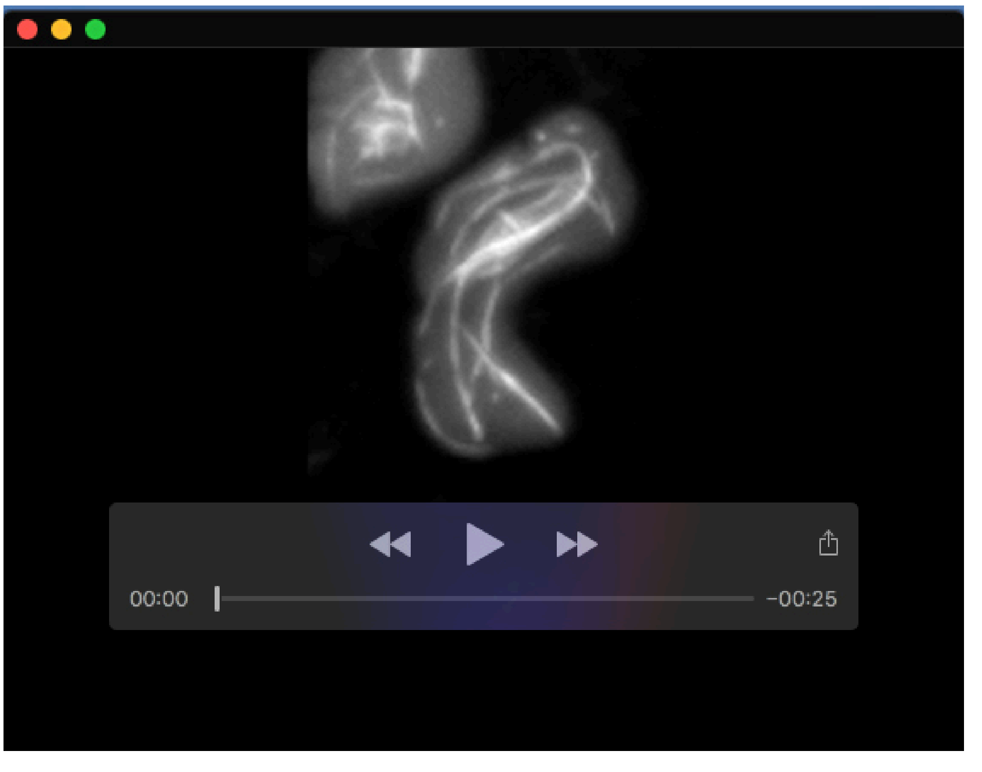

Movie 7. Nuclear F-actin dynamics in Lamin A knockdown HeLa cells expressing actinWT-NLS and nAC-GFP. G1-synchronized HeLa cells were seeded on glass-bottom dishes, co-transfected with siRNA against Lamin A and expression plasmids for actinWT-NLS and nAC-GFP, and imaged live by confocal microscopy 20 hours after transfection. A 1.5-hour timelapse was acquired, imaging every 10 minutes. The yellow arrowhead in the first frame indicates a site of actin accumulation at the nuclear rim, and only interphase is shown.

Table S1. Raw data for all experiments.

Click here to download Table S1

Journal of Cell Science • Supplementary information
