# Supplementary material for: Nuclear F-actin and Lamin A antagonistically modulate nuclear shape
Source: J Cell Sci. 2022 Jul 4;135(13):jcs259692. doi: 10.1242/jcs.259692 (PMC9377710; doi:10.1242/jcs.259692)
Supplement: Supplementary file 1 [file joces-135-259692_review_history.pdf]

Nuclear F-actin and Lamin A antagonistically modulate nuclear shape

Sampada Mishra and Daniel L. Levy
DOI: 10.1242/jcs.259692

Editor: Maria Carmo-Fonseca

Review timeline

Original submission:	15 December 2021
Editorial decision:	31 January 2022
First revision received:	4 May 2022
Editorial decision:	23 May 2022
Second revision received:	24 May 2022
Accepted:	28 May 2022

Original submission

First decision letter

MS ID#: JOCES/2021/259692

MS TITLE: Nuclear F-actin and lamin A antagonistically modulate nuclear shape

AUTHORS: Sampada Mishra and Daniel L. Levy
ARTICLE TYPE: Research Article

We have now reached a decision on the above manuscript.

To see the reviewers' reports and a copy of this decision letter, please go to: https://submit-jcs.biologists.org and click on the 'Manuscripts with Decisions' queue in the Author Area. (Corresponding author only has access to reviews.)

As you will see, the reviewers raise a number of substantial criticisms that prevent me from accepting the paper at this stage. They suggest, however, that a revised version might prove acceptable, if you can address their concerns. If you think that you can deal satisfactorily with the criticisms on revision, I would be pleased to see a revised manuscript. We would then return it to the reviewers.

We are aware that you may be experiencing disruption to the normal running of your lab that makes experimental revisions challenging. If it would be helpful, we encourage you to contact us to discuss your revision in greater detail. Please send us a point-by-point response indicating where you are able to address concerns raised (either experimentally or by changes to the text) and where you will not be able to do so within the normal timeframe of a revision. We will then provide further guidance. Please also note that we are happy to extend revision timeframes as necessary.

Please ensure that you clearly highlight all changes made in the revised manuscript. Please avoid using 'Tracked changes' in Word files as these are lost in PDF conversion.

I should be grateful if you would also provide a point-by-point response detailing how you have dealt with the points raised by the reviewers in the 'Response to Reviewers' box. Please attend to

© 2022. Published by The Company of Biologists under the terms of the Creative Commons Attribution License (https://creativecommons.org/licenses/by/4.0/).

all of the reviewers' comments. If you do not agree with any of their criticisms or suggestions please explain clearly why this is so.

Reviewer 1

Advance summary and potential significance to field

In the manuscript entitled "Nuclear F-actin and lamin A antagonistically modulate nuclear shape" the authors developed an assay to assemble nuclei in Xenopus egg extracts in the presence of nuclear F-actin. The authors describe the formation of a biolobed nucleus that is dependent on Lamin-A and nuclear actin nucleation by formins. The data suggesting a role for formins on nuclear shape, probably through its effect on nuclear actin, is of interest. However the observed phenotype might be specific for in vitro nuclear formation in the xenopus extracts in the presence of F-actin, as described in detail below.The findings described using HeLa cells are not novel and were described in Lamm et al NCB 2020 and baarlink et al NCB 2017. Therefore, although technically the manuscript is very well organized, important points must be addressed for publication

Comments for the author

Major point:
Is there any evidence that the nuclear f-actin observed in Xenopus extracts formed nuclei to behave as nuclear actin observed in mammalian cells, or in any other cells? Usually, nuclear actin is more rod-like shape, (see for instance Baarlink et al NCB 2017 or Lamm et al. NCB 2020). Also, in mammalian cells nuclear cortical actin has never been observed to the best of my knowledge. Therefore is the nuclear cortical actin observed in the Xenopus extracts also occurring in the nucleus of cells? Finally, the dramatic asymmetry of NPC chromatin (Fig 2) and LaminB3 (supp figure 1B3) is not observed in the nucleus of cells, both in the current manuscript (figure 5) and published work (Lamm et al NCB 2020, Baarlink et al NCB 2017). The bilobes devoid of chromatin and NPC resembled nuclear blebs observed during cell migration through constriction (Rabb et al. Science 2016; Deanis et al. Science 2016). Therefore, all these observations raise the possibility that the observed role of F-actin in the nucleus is specific to the used in vitro system, and not occurring in the nuclei of cells. It is therefore important that the authors provide a rational and experimental evidence supporting that the in vitro system they are using with f-actin inside the nucleus mimics what is observed in cells.
In the abstract and conclusion, the authors should not mention that their data suggest that nuclear F-actin generate an intranuclear actin ring, since this is not shown in cells, only in nuclei formed in the in vitro xenopus extract.

Minor points:
Page 6, fig 3A-D. the authors should provide statistical information (number of cells measured, number of independent experiments) regarding the quantification of nuclei with actin inside the nucleus (65%) and only one peak (35%). Also the authors should tone down their conclusion in this paragraph since these data does not support on its own that nuclear F-actin might be responsible for bilobed nuclear morphology.
Figure 4A – upon addition of Lamin A, the amount of nuclear cortical actin/actin rim seems to be reduced. It would be informative and important for the proposed model that the authors quantify the actin rim as they did in figure 2C.
Figure 4A-B. Panel A is a representative example of cells quantified in panel B?
if so, then it would be nice to indicate in fig 4B which data point corresponds to the image in figure 4A so the reader has a better idea how representative is the image.
Fig 5E-F should become Fig 5C-D since they are mentioned before in the main text.
In the experiments using Hela cells (fig 5 and supp figure 4), in the conditions where lobulation is exarcebated, or nuclei are multilobulated, how do the authors know that these defects are not caused by defects in mitosis that give rise to multinuclear cells, micronuclei or fragmented nuclei?
Sup Fig 5A – in the figure legend it is mentioned that CK-636 was used at 100uM or 200uM, however in the figure, only one condition is showed.
Sup Fig 5A -B- CK-666 was used at a concentration of 500uM (fig &E) and CK-869 was used at a concentration of 0.1uM to 50uM. Both inhibitors have a similar IC50 of 7 uM (Nolen et al. Nature 2009). Is there a reason for the use of CK-666 at a 10x higher concentration than CK-869?

© 2022. Published by The Company of Biologists under the terms of the Creative Commons Attribution License (https://creativecommons.org/licenses/by/4.0/).

Reviewer 2

Advance summary and potential significance to field

Using cell free Xenopus egg extracts and HeLa cells the authors present data that suggesting that excessive nuclear F-actin formation can distort the nuclear envelope, an effect which can be counteracted by lamin A. The manuscript is interesting and well written. Given the importance of nuclear envelope malformation in pathologies (laminopathies and certain cancers) the factors and mechanisms stabilizing or destabilizing the nuclear envelope and acting against each other are of high interest. Here, the work suggest that nuclear F-actin and lamin A have such opposite functions.

Comments for the author

1) Figs 1, 2, 4, 5 and 6 as well as S2, S5 show plots with several modalities of statistical analysis applied to different data sets; In the methods section it is clearly reported how it is determined which statistical analysis is applied to each case. The Number of data points per condition is also indicated in the figure legends. However, there is no indication about the number of independent experiments (biological replicates). It would be convenient for reproducibility and robustness analysis to indicate the number of biological replicates and apply plotting and statistical analysis following the recommendations of Lord et al. 2020 (https://doi.org/10.1083/jcb.202001064) and Goedhart 2020 (https://doi.org/10.1091/mbc.E20-09-0583). In addition, the legends of these figures indicate that the error bars on the plots indicate the 95% CI. This means that there is only a 5% chance that a true value is NOT included within the span error bars. The narrow bar span at some of the data sets with very large dispersions, might indicate that an error in the description and SEs are shown. For the current plots in the manuscript, SD bars would be recommended.
2) In Fig 1, samples treated with 1 ng/ul Cytochalasin B clearly show filamentous actin structures in the nucleoplasm, which are not visible in DMSO or higher Cytochalasin B concentrations. Indeed, these nucleoplasmic filaments (not the membrane ring) are observed in lobulated nuclei in both, DNA and bleb compartment in figs 2 and 3. This could indicate that the addition of Cytochalasin B at low concentration does not prevent F-acting formation in nucleoplasm but actin nucleation/polymerization around the nuclear envelope.
This might be a different process or just a by-product of an excess id F-Actin in absence of Lamins (or unbalanced stoichiometry). These considerations would affect the mechanistic explanation of the phenotype.
3) In the plots and in the methods section it is not quite clear how the segmentation has been performed in order to extract the intensity values.
Furthermore, if the intensity values have been extracted from fluorescence-wide field images (the ones shown seem to be) the out of focus light from the nuclear rim could impact the measurements. Therefore, it is difficult to make a firm conclusion. I recommend to clearly describe the segmentation procedure to measure the intensity values as well as to indicate when the measurements have been done from wide field images and when from confocal. In the latter case, at least the pinhole information would be necessary.
3) In the figure 5, images and circularity plots are used to describe the phenotypes. However, in the text, most of the explanations are based on percentages of cells under an arbitrary circularity threshold of 0.6. In the current form, the plots do not help to appreciate this percentage values. For clarity and robustness, it would be convenient to present them separately in dedicated plots with the adequate statistical test (Fisher Test or chi-squared test).
4.) So far, the phenotype in cells is only severe after artificial accumulation of active actin in the nucleus. In fact, no analysis of nuclear actin in cells in the different experimental conditions has been shown. For example, to clarify the function of native actin dynamics in cell nuclei morphology (and compare with Xenopus system), live imaging with LifeAct-GFP in cells in the conditions of the figure 5 and 7 could be performed.
4) In which part of the cell cycle appear the phenotypes in siLMNA + ActinWT-NLS cells? Do they appear in cells in interphase nuclei or are they generated during post-mitotic nuclear reassembly and G1?. Is mitotic progression normal in siLMNA + ActinWT-NLS cells? The images of multitoned nuclei in the S4 figure are consistent with severe defects on chromosome segregation during mitosis.
5) In the text it is said, “inhibiting formins with SMIFH2 in F-actin intact extract reduced the staining intensity of nucleoplasmic and rimlocalized F-actin

© 2022. Published by The Company of Biologists under the terms of the Creative Commons Attribution License (https://creativecommons.org/licenses/by/4.0/).

(Fig 6A-6C)”. Like for the Cytochalasin B experiments (comment 2), the images suggest a diminution of the actin signal at the nuclear cortex but not sure at the nucleoplasm.
6) Due to the difference of sharpness and resolution from the images A-B vs C in figure S1, it is not clear whether the images proceed from a fluorescence-widefield or confocal microscope or both. This information is necessary in order to extract reasonable conclusions from staining distributions in microscopic volumes.
7) In the discussion the authors comment about “What determines the site of nuclear lobe formation?”. It would be worth to elaborate here about the discrepancy of DNA distribution in the malformed nuclei form Xenopus experiments and HeLa cells. In the same line, the cortical ring of actin present in the in vitro assays. Some efforts should be done to test the actin distribution in nuclei in the cell experiments context of this work, or at least comment on absence of F-actin at the inner nuclear envelope in the cells shown on Grosse´s Lab works (Krippner 2020; Wang 2019; Plessner 2015…)

Minor points:
8.) It would be interesting to visualize lamin A/C in the experiments performed in Fig 4. Does it localize to the nuclear envelope? Does this sow a similarly uneven distribution as NPCs and lamin B?
9) figure legend 5. Although the figure is largely self-explanatory the sentence “(A) Representative images are shown for overexpression of nuclear targeted actin and/or lamin A knockdown” is formally not correct as the panel also includes pictures showing control RNAi and mCherry only. Same applies to (C).
10.) I suggest switching panel C/D and panel E/F in figure 5 to follow the flow of the text.
11.) Given that fertilized Xenopus eggs lack lamin A/C it remains open what in the first embryonic cell cycles prevents nuclear actin from generating bi-lobuled nuclei in the natural situation. In other words, what takes the function of cytochalasin B in the early developing tadpoles? This might be worth to discuss or address in future studies.

First revision

Author response to reviewers' comments

Response to reviews

Reviewer comments are copied in *italics*. Our responses follow below each reviewer comment.

Reviewer 1 Advance Summary and Potential Significance to Field:
In the manuscript entitled “Nuclear F-actin and lamin A antagonistically modulate nuclear shape” the authors developed an assay to assemble nuclei in Xenopus egg extracts in the presence of nuclear F-actin. The authors describe the formation of a biolobed nucleus that is dependent on Lamin-A and nuclear actin nucleation by formins. The data suggesting a role for formins on nuclear shape, probably through its effect on nuclear actin, is of interest. However, the observed phenotype might be specific for in vitro nuclear formation in the xenopus extracts in the presence of F-actin, as described in detail below. The findings described using HeLa cells are not novel and were described in Lamm et al NCB 2020 and baarlink et al NCB 2017.

This is not strictly correct. The Baarlink paper primarily uses NIH3T3 cells and mouse embryos, not HeLa cells. The Lamm paper primarily uses U2OS and IMR90 cells, not HeLa cells. A key aspect of our cell culture experiments is examining nuclear shape alterations in lamin A knockdown cells. While Baarlink did examine nuclear actin filaments upon lamin A knockdown, that paper did not address the contribution of nuclear actin filaments to nuclear shape alterations induced upon lamin A knockdown. Lamm performed no lamin A knockdown experiments. In our paper we report on the contribution of formins to nuclear shape changes induced by lamin A knockdown. The cited Baarlink and Lamm papers do not examine formins. Lastly, while our paper primarily deals with nuclear shape changes, Baarlink highlights the contribution of nuclear F-actin to nuclear expansion after mitotic exit and Lamm focuses on the role of nuclear F-actin in the DNA replication stress response. Thus, in addition to our novel *Xenopus* studies, we feel our cell culture experiments also make unique contributions, particularly with respect to the regulation of nuclear shape.

© 2022. Published by The Company of Biologists under the terms of the Creative Commons Attribution License (https://creativecommons.org/licenses/by/4.0/).

Therefore, although technically the manuscript is very well organized, important points must be addressed for publication.

Reviewer 1 Comments for the Author:
Major point:
Is there any evidence that the nuclear f-actin observed in Xenopus extracts formed nuclei to behave as nuclear actin observed in mammalian cells, or in any other cells? Usually, nuclear actin is more rod-like shape, (see for instance Baarlink et al NCB 2017 or Lamm et al. NCB 2020). Also, in mammalian cells, nuclear cortical actin has never been observed to the best of my knowledge. Therefore is the nuclear cortical actin observed in the Xenopus extracts also occurring in the nucleus of cells?

While most instances of nuclear F-actin in these papers appear rod-like, there are also examples where some accumulation of actin at the nuclear rim is evident. Examples include:
Baarlink - Fig. 1A (time point 21:00), Fig. 2A, Fig. 3A (time point 27:30), Fig. 4A (later time points), Fig. 6F, Fig. S6E
Lamm - Fig. S1J (0:05), Fig. S1K (nucleus on the far right), Fig. S3H (nucleus on the far right).

This suggests that nuclear rim accumulation of actin may be a normal occurrence in cells that is particularly enhanced in our *Xenopus* nuclei. To confirm this, we performed fixed and live imaging in HeLa cells transfected with a GFP-tagged nuclear actin chromobody (nAC-GFP), the same probe used in the Baarlink and Lamm papers. In a subset of nuclei, we observe accumulation of actin at the nuclear rim. This is now briefly mentioned in the text and the data are presented in Fig. S6B and Movies 4-7.

There are other cell types where intranuclear rim localization of actin has been reported. In starfish oocytes, an F-actin shell forms at the nuclear envelope to promote nuclear envelope breakdown (PMID 24909322, 29350616, 31989921). Similar F-actin nuclear shells have been observed in a variety of other species including early sea urchin and sand dollar embryos, *Nematostella vectensis*, and polychaete worms (PMID 17685442, 25433655, 10213079). F-actin is also observed at the nuclear rim in *Xenopus laevis* oocytes (PMID 16489345) and mouse oocytes (PMID 35112707) as well as in mesenchymal stem cells (PMID 31648392) and several cultured mammalian cell lines (PMID 23558171). These observations support the idea that the intranuclear rim of actin we observe is not an artifact of the *Xenopus* extract system. Rather, the prominence of this structure may depend on cell type, species, developmental stage, and/or disease state. For example, accumulation of actin at the intranuclear rim may be a characteristic of nuclei in oocytes and early stage embryos. That could explain why we observe this actin structure in egg extract, indicating the extract system is recapitulating a normal feature of oocyte nuclei. We argue that this actin structure is indeed physiologically relevant, at least in the context of early development, underscoring the need to examine this structure in other cell types. We now comment on this idea in the manuscript.

Finally, the dramatic asymmetry of NPC, chromatin (Fig 2) and LaminB3 (supp figure 1B3) is not observed in the nucleus of cells, both in the current manuscript (figure 5) and published work (Lamm et al NCB 2020, Baarlink et al NCB 2017). The bilobes devoid of chromatin and NPC resembled nuclear blebs observed during cell migration through constriction (Rabb et al. Science 2016; Deanis et al. Science 2016).

This is an interesting point that we have now examined more closely using SoRa super- resolution microscopy. In a subset of siLMNA+actinWT-NLS transfected cells with aberrant nuclei, we do observe asymmetric distribution of lamin B1 and NPCs.
Generally, areas of reduced chromatin amount correlate with reduced lamin B1 and NPC staining. Furthermore, we also note instances where actin enrichment at the nuclear rim correlates with reduced lamin B1 and chromatin staining. These data are now described in the manuscript and presented in Fig. S6. We also cite published studies where lamin A knockdown or mutation leads to similar asymmetric distributions of nuclear envelope proteins (PMID 10579712 and 25738644). Lamm and Baarlink may have not observed this asymmetric distribution of nuclear envelope markers because they did not focus on lamin A knockdown cells. We now note in the manuscript that the structure of the bilobed nuclei we observed in extract are reminiscent of nuclear blebs that form during confined cell migration, citing the papers mentioned.

© 2022. Published by The Company of Biologists under the terms of the Creative Commons Attribution License (https://creativecommons.org/licenses/by/4.0/).

Therefore, all these observations raise the possibility that the observed role of F-actin in the nucleus is specific to the used in vitro system, and not occurring in the nuclei of cells. It is therefore important that the authors provide a rational and experimental evidence supporting that the in vitro system they are using with f-actin inside the nucleus mimics what is observed in cells.

We have taken a closer look at nuclear actin in Xenopus nuclei through time-lapse imaging. Soon after nuclear assembly, we observe nucleoplasmic actin filaments and rods that are reminiscent of what is observed in cell culture. At later time points we observe enrichment of actin at the nuclear rim as nuclear growth leads to bilobe formation. These data are now mentioned in the text and presented in Movies 2 and 3. Something similar may be happening over time in the Baarlink and Lamm examples noted above where some nuclear rim accumulation of actin is noted. This suggests that what we observe in the Xenopus system is not that different from what is observed in cells.

We have also acquired time-lapse images of nuclear actin in HeLa cells using nAC- GFP, as noted above. We observe different types of nuclear actin structures, including complete or partial lining of the nuclear rim with actin, and these data are now mentioned in the text and shown in Fig. S6B and Movies 4-7. In some time-lapses we observe a behavior similar to that seen in Xenopus nuclei, where initial formation of smaller actin filaments and rods transitions to longer filaments with partial accumulation at the nuclear rim (Movies 2 and 3). Thus, we argue that at least a subset of HeLa cells display actin structures and dynamics that are reminiscent of what we observe in vitro. Perhaps there is a greater tendency for actin filaments to accumulate at the nuclear rim in Xenopus nuclei because of their larger size or because of differences in actin dynamics.

In the abstract and conclusion, the authors should not mention that their data suggest that nuclear F-actin generate an intranuclear actin ring, since this is not shown in cells, only in nuclei formed in the in vitro xenopus extract.

This is a valid point, although we do now present some evidence for accumulation of actin at the nuclear rim in HeLa cells and discuss studies where an intranuclear actin shell was described for other species. We have now toned down this conclusion in the abstract and discussion. If necessary, we are willing to deemphasize the whole issue and simply state that nuclear actin is present in Xenopus extract nuclei and affects nuclear morphology. Presenting our current understanding of the structure of Xenopus nuclear actin lays the groundwork for future studies to generate more precise structural details.

Minor points:

Page 6, fig 3A-D. the authors should provide statistical information (number of cells measured, number of independent experiments) regarding the quantification of nuclei with actin inside the nucleus (65%) and only one peak (35%). Also the authors should tone down their conclusion in this paragraph since these data does not support on its own that nuclear F-actin might be responsible for bilobed nuclear morphology.

We have added the requested information to the Figure Legend and toned down the conclusion.

Figure 4A – upon addition of Lamin A, the amount of nuclear cortical actin/actin rim seems to be reduced. It would be informative and important for the proposed model that the authors quantify the actin rim as they did in figure 2C.

We have added this quantification to Fig. S3A and comment on the result in the text.

Figure 4A-B. Panel A is a representative example of cells quantified in panel B? if so, then it would be nice to indicate in fig 4B which data point corresponds to the image in figure 4A so the reader has a better idea how representative is the image.
The measured circularities for the representative nuclei shown are now noted in the legend.

Fig 5E-F should become Fig 5C-D since they are mentioned before in the main text.

The panel order has been corrected.

© 2022. Published by The Company of Biologists under the terms of the Creative Commons Attribution License (https://creativecommons.org/licenses/by/4.0/).

In the experiments using Hela cells (fig 5 and supp figure 4), in the conditions where lobulation is exarcebated, or nuclei are multilobulated, how do the authors know that these defects are not caused by defects in mitosis that give rise to multinuclear cells, micronuclei or fragmented nuclei?

Reviewer 2 had a similar question and we have now performed extensive experimentation to address when in the cell cycle the changes in nuclear morphology occur.

Our first approach was to attempt cell cycle synchronization. HeLa cells were simultaneously transfected and treated with lovastatin to induce G1 arrest, followed by release into the cell cycle using mevalonic acid. By fixing cells at different time points after release we hoped to identify when in the cell cycle changes in nuclear morphology occur. This was unsuccessful because cells did not tolerate simultaneous transfection and lovastatin treatment.

Next, we altered the protocol slightly by first transfecting cells with siLMNA+actinWT- NLS and waiting 12 hours before treating with lovastatin. 36 hours post-transfection the cells were arrested in G1, something we confirmed by quantifying Hoechst staining intensity. Cells were either maintained in lovastatin for another 12 hours or released into S phase. We wanted to determine if nuclear circularity decreased as cells progressed through interphase. However, we found that the nuclear shape change had already occurred 36 hours post-transfection (Fig. R1). Though not definitive, this experiment suggested that some change in nuclear shape might occur during G1.

We also tried pre-synchronizing the cells with lovastatin, releasing them into the cell cycle, and then transfecting. As observed before, nuclear shape changes had already occurred 36 hours post-transfection (Fig. R2).

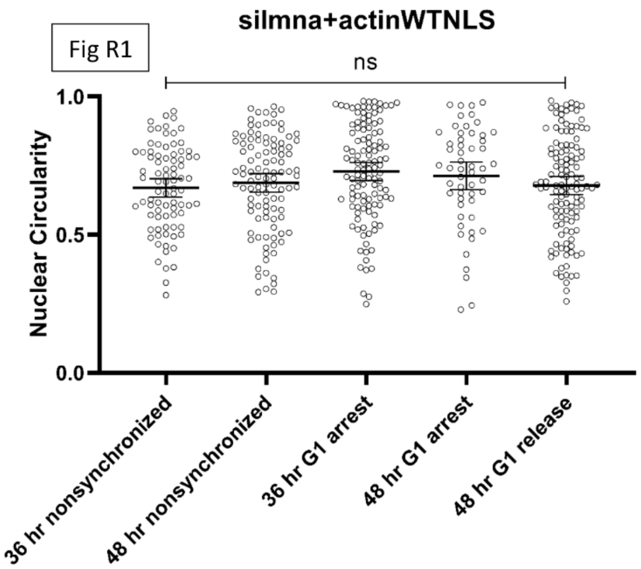

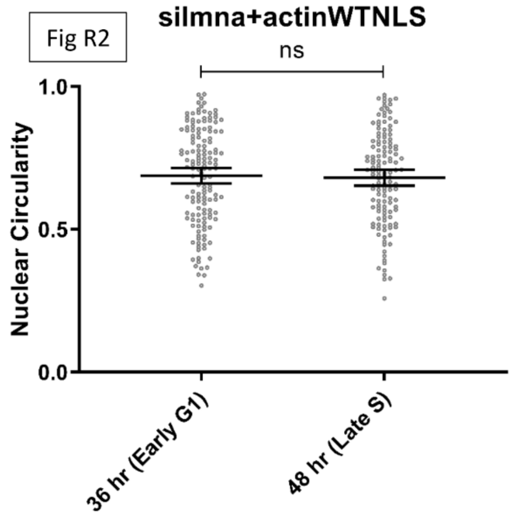

We next turned to live imaging at earlier time points after transfection. We synchronized H2B-GFP tagged HeLa cells in G1 with lovastatin, released them into the cell cycle, transfected with siLMNA+actinWT-NLS, and imaged live for up to 60 hours post- transfection. We imaged H2B-GFP to assess when after mitosis nuclear shape changes occurred. Based on data from three independent experiments, we classified the nuclei into four categories: no shape change, shape change in G1 following a normal mitosis, shape change in G1 due to mitotic defects, and shape change in S/G2 (i.e. nucleus changes from round to misshapen, lobulated, or elongated). While some instances of mitotic defects were observed, the majority of nuclei exhibited shape changes during interphase. These new data are now presented in Fig. S7 and discussed in the text.

Lastly, since inhibiting formins for only one hour was sufficient to largely rescue the nuclear shape defects resulting from Lamin A knockdown, this suggests that dynamic F- actin is required to maintain abnormal nuclear shape and that passage through mitosis was not required to rescue nuclear morphology (Fig. 7).

© 2022. Published by The Company of Biologists under the terms of the Creative Commons Attribution License (https://creativecommons.org/licenses/by/4.0/).

Sup Fig 5A – in the figure legend it is mentioned that CK-636 was used at 100uM or 200uM, however in the figure, only one condition is showed.

Because both concentrations yielded results similar to the control, we grouped the data from the two concentrations together. In the revised manuscript we now present the data for only one concentration to simplify the message.

Sup Fig 5A -B- CK-666 was used at a concentration of 500uM (fig &E) and CK-869 was used at a concentration of 0.1uM to 50uM. Both inhibitors have a similar IC50 of 7 uM (Nolen et al. Nature 2009). Is there a reason for the use of CK-666 at a 10x higher concentration than CK-869?

It is not uncommon that higher drug concentrations are required to be effective in Xenopus egg extract compared to intact cells. This may be because inhibitors are rendered ineffective in extract when absorbed into abundant membrane compartments and/or because active transport in cells results in higher intracellular concentrations of inhibitors (PMID 28982779). This is the reason why we used the higher concentration of CK-666 in extract. On the other hand, CK-869 has been shown to alter interphase microtubule asters and prevent nuclear assembly at concentrations greater than 50 µM, explaining why we used lower concentrations of that inhibitor (PMID 29395083).

Reviewer 2 Advance Summary and Potential Significance to Field:
Using cell free Xenopus egg extracts and HeLa cells the authors present data that suggesting that excessive nuclear F-actin formation can distort the nuclear envelope, an effect which can be counteracted by lamin A. The manuscript is interesting and well written. Given the importance of nuclear envelope malformation in pathologies (laminopathies and certain cancers) the factors and mechanisms stabilizing or destabilizing the nuclear envelope and acting against each other are of high interest. Here, the work suggest that nuclear F-actin and lamin A have such opposite functions.

Reviewer 2 Comments for the Author:
1) Figs 1, 2, 4, 5 and 6 as well as S2, S5 show plots with several modalities of statistical analysis applied to different data sets; In the methods section it is clearly reported how it is determined which statistical analysis is applied to each case. The Number of data points per condition is also indicated in the figure legends. However, there is no indication about the number of independent experiments (biological replicates). It would be convenient for reproducibility and robustness analysis to indicate the number of biological replicates and apply plotting and statistical analysis following the recommendations of Lord et al. 2020 (https://doi.org/10.1083/jcb.202001064) and Goedhart 2020 (https://doi.org/10.1091/mbc.E20-09-0583).

We have now added the number of biological replicates to each figure legend.

In addition, the legends of these figures indicate that the error bars on the plots indicate the 95% CI. This means that there is only a 5% chance that a true value is NOT included within the span error bars. The narrow bar span at some of the data sets with very large dispersions, might indicate that an error in the description and SEs are shown. For the current plots in the manuscript, SD bars would be recommended.

From the Graphpad website: “The 95% confidence interval defines a range of values that you can be 95% certain contains the population mean. With large samples, you know that mean with much more precision than you do with a small sample, so the confidence interval is quite narrow when computed from a large sample.” So, 95% confidence intervals indicate that there is only a 5% chance that the true MEAN is not included within the span error bars, not that a true value is not included within the span error bars. Our relatively large sample sizes explain why the confidence intervals are quite narrow.

We prefer to present confidence intervals rather than SD. Because we plot the raw data, the variance in our data is already clearly shown in a more intuitive manner than with SD. We feel plotting the raw data and 95% confidence intervals is easier for most readers to understand, rather than SD which is less intuitive for many (including myself). In the rare instances where we plot bar graphs instead of individual data points, SD error bars are shown (e.g. Fig. S5B).

© 2022. Published by The Company of Biologists under the terms of the Creative Commons Attribution License (https://creativecommons.org/licenses/by/4.0/).

That being said, we now include Table S1 with all of our raw data, including calculation of SDs.

2) In Fig 1, samples treated with 1 ng/ul Cytochalasin B clearly show filamentous actin structures in the nucleoplasm, which are not visible in DMSO or higher Cytochalasin B concentrations. Indeed, these nucleoplasmic filaments (not the membrane ring) are observed in lobulated nuclei in both, DNA and bleb compartment in figs 2 and 3. This could indicate that the addition of Cytochalasin B at low concentration does not prevent F-actin formation in nucleoplasm but actin nucleation/polymerization around the nuclear envelope. This might be a different process or just a by-product of an excess id F-Actin in absence of Lamins (or unbalanced stoichiometry). These considerations would affect the mechanistic explanation of the phenotype.

This is an interesting point. We now include quantification of nucleoplasmic F-actin (excluding the rim signal) in Fig. S2A. Based on these data, we mention that nucleoplasmic F-actin filaments are visible and that experiments with low cytochalasin concentrations suggest that rim-localized F-actin may be more sensitive to depolymerization than nucleoplasmic F-actin. In the Discussion we present two possible models for bleb formation: F-actin nucleation at the nuclear rim vs. F-actin that nucleates in the nucleoplasm and then moves to the rim.

3) In the plots and in the methods section it is not quite clear how the segmentation has been performed in order to extract the intensity values. Furthermore, if the intensity values have been extracted from fluorescence-wide field images (the ones shown seem to be) the out of focus light from the nuclear rim could impact the measurements. Therefore, it is difficult to make a firm conclusion. I recommend to clearly describe the segmentation procedure to measure the intensity values as well as to indicate when the measurements have been done from wide field images and when from confocal. In the latter case, at least the pinhole information would be necessary.

We now indicate in the Figure Legends when imaging and quantification were performed for wide field versus confocal images. The confocal pinhole information and more detailed descriptions of our intensity quantification approaches have been added to the Methods section. All HeLa cell data were acquired by confocal imaging. While much of the imaging of nuclei in Xenopus extract was by wide-field, NPC and lamin B3 intensity measurements were from confocal images. The only other intensity measurements we performed for nuclei assembled in Xenopus extract were for actin intensity, and in general the observed differences were quite large and appeared to be accurately reflected in our quantitative measurements. Side-by-side measurements of nuclear actin intensities from wide field and confocal images showed similar trends.

4) In the figure 5, images and circularity plots are used to describe the phenotypes. However, in the text, most of the explanations are based on percentages of cells under an arbitrary circularity threshold of 0.6. In the current form, the plots do not help to appreciate this percentage values. For clarity and robustness, it would be convenient to present them separately in dedicated plots with the adequate statistical test (Fisher Test or chi-squared test).

We have added the requested plots and statistical analyses in Fig. S5B.

5.) So far, the phenotype in cells is only severe after artificial accumulation of active actin in the nucleus. In fact, no analysis of nuclear actin in cells in the different experimental conditions has been shown. For example, to clarify the function of native actin dynamics in cell nuclei morphology (and compare with Xenopus system), live imaging with LifeAct-GFP in cells in the conditions of the figure 5 and 7 could be performed.

Reviewer 1 asked a similar question. We chose to use a GFP-tagged nuclear actin chromobody (nAC-GFP) rather than LifeAct-GFP so our data would be more directly comparable to published studies cited by Reviewer 1. We have now performed fixed imaging in lamin A knockdown HeLa cells expressing nuclear-targeted actin and nAC- GFP. In a subset of nuclei we observe accumulation of actin at the nuclear rim, and this is now briefly mentioned in the text and the data are presented in Fig. S6. We have also acquired time-lapse images of nuclear actin in HeLa cells using nAC-GFP. We observe different types of nuclear actin structures, including complete or partial lining of the nuclear rim with actin, and these data are now mentioned in the text and shown in Fig. S6B and Movies 4-7. In some time-lapses we observe a behavior similar to that seen in Xenopus nuclei, where initial formation of smaller actin filaments and rods transitions to longer

© 2022. Published by The Company of Biologists under the terms of the Creative Commons Attribution License (https://creativecommons.org/licenses/by/4.0/).

filaments with partial accumulation at the nuclear rim (Movies 2 and 3). Thus we argue that at least a subset of HeLa cells display actin structures and dynamics that are reminiscent of what we observe in vitro. Perhaps there is a greater tendency for actin filaments to accumulate at the nuclear rim in Xenopus nuclei because of their larger size or because of differences in actin dynamics.

6) In which part of the cell cycle appear the phenotypes in siLMNA + ActinWT-NLS cells? Do they appear in cells in interphase nuclei or are they generated during post- mitotic nuclear reassembly and G1?. Is mitotic progression normal in siLMNA + ActinWT-NLS cells? The images of multitoned nuclei in the S4 figure are consistent with severe defects on chromosome segregation during mitosis.

We have now performed extensive experimentation to address when in the cell cycle the changes in nuclear morphology occur.

Our first approach was to attempt cell cycle synchronization. HeLa cells were simultaneously transfected and treated with lovastatin to induce G1 arrest, followed by release into the cell cycle using mevalonic acid. By fixing cells at different time points after release we hoped to identify when in the cell cycle changes in nuclear morphology occur. This was unsuccessful because cells did not tolerate simultaneous transfection and lovastatin treatment.

Next, we altered the protocol slightly by first transfecting cells with siLMNA+actinWT- NLS and waiting 12 hours before treating with lovastatin. 36 hours post-transfection the cells were arrested in G1, something we confirmed by quantifying Hoechst staining intensity. Cells were either maintained in lovastatin for another 12 hours or released into S phase. We wanted to determine if nuclear circularity decreased as cells progressed through interphase. However, we found that the nuclear shape change had already occurred 36 hours post-transfection (Fig. R1). Though not definitive, this experiment suggested that some change in nuclear shape might occur during G1.

We also tried pre-synchronizing the cells with lovastatin, releasing them into the cell cycle, and then transfecting. As observed before, nuclear shape changes had already occurred 36 hours post-transfection (Fig. R2).

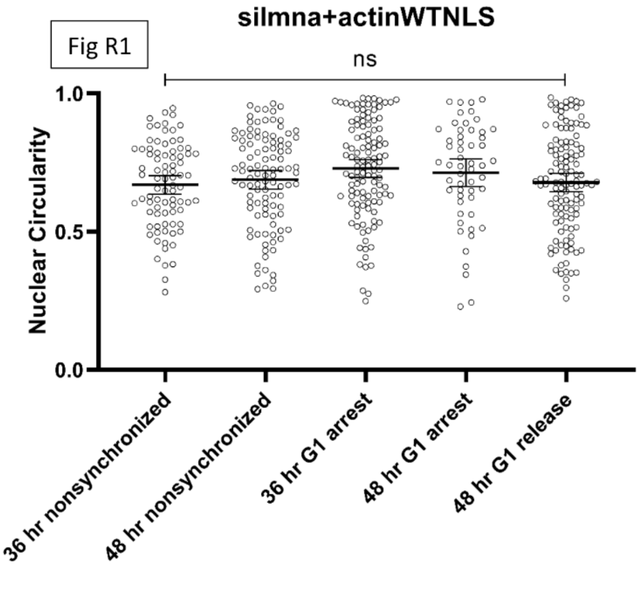

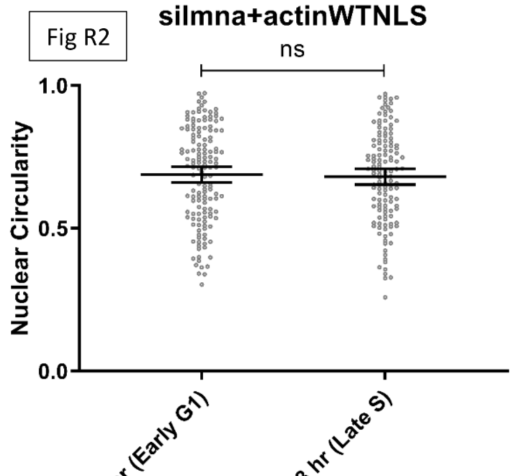

We next turned to live imaging at earlier time points after transfection. We synchronized H2B-GFP tagged HeLa cells in G1 with lovastatin, released them into the cell cycle, transfected with siLMNA+actinWT-NLS, and imaged live for up to 60 hours post- transfection. We imaged H2B-GFP to assess when after mitosis nuclear shape changes occurred. Based on data from three independent experiments, we classified the nuclei into four categories: no shape change, shape change in G1 following a normal mitosis, shape change in G1 due to mitotic defects, and shape change in S/G2 (i.e. nucleus changes from round to misshapen, lobulated, or elongated). While some instances of

© 2022. Published by The Company of Biologists under the terms of the Creative Commons Attribution License (https://creativecommons.org/licenses/by/4.0/).

mitotic defects were observed, the majority of nuclei exhibited shape changes during interphase. These new data are now presented in Fig. S7 and discussed in the text.

Lastly, since inhibiting formins for only one hour was sufficient to largely rescue the nuclear shape defects resulting from Lamin A knockdown, this suggests that dynamic F- actin is required to maintain abnormal nuclear shape and that passage through mitosis was not required to rescue nuclear morphology (Fig. 7).

7) In the text it is said, "inhibiting formins with SMIFH2 in F-actin intact extract reduced the staining intensity of nucleoplasmic and rimlocalized F-actin (Fig 6A-6C)". Like for the Cytochalasin B experiments (comment 2), the images suggest a diminution of the actin signal at the nuclear cortex but not sure at the nucleoplasm.

We have now quantified the nucleoplasmic F-actin signal (excluding the rim signal) and these data are presented in Figs. S2A and S3B.

8) Due to the difference of sharpness and resolution from the images A-B vs C in figure S1, it is not clear whether the images proceed from a fluorescence-widefield or confocal microscope or both. This information is necessary in order to extract reasonable conclusions from staining distributions in microscopic volumes.

We now indicate in the Figure Legends when imaging and quantification were performed for wide field versus confocal images.

9) In the discussion the authors comment about "What determines the site of nuclear lobe formation?". It would be worth to elaborate here about the discrepancy of DNA distribution in the malformed nuclei form Xenopus experiments and HeLa cells.

Reviewer 1 raised the same question and we have now examined this issue more closely in HeLa cells using SoRa super-resolution microscopy. In a subset of siLMNA+actinWT-NLS transfected cells with aberrant nuclei, we do observe asymmetric distribution of lamin B1 and NPCs. Generally, areas of reduced chromatin amount correlate with reduced lamin B1 and NPC staining. Furthermore, we also note instances where actin enrichment at the nuclear rim correlates with reduced lamin B1 and chromatin staining. These data are now described in the manuscript and presented in Fig. S6. We also cite published studies where lamin A knockdown or mutation leads to similar asymmetric distributions of nuclear envelope proteins and chromatin (PMID 10579712 and 25738644).

In the same line, the cortical ring of actin present in the in vitro assays. Some efforts should be done to test the actin distribution in nuclei in the cell experiments context of this work, or at least comment on absence of F-actin at the inner nuclear envelope in the cells shown on Grosse´s Lab works (Krippner 2020; Wang 2019; Plessner 2015…)

As noted above, we now include new data on the distribution of nuclear F-actin in our HeLa cell experiments in Fig. S6 and Movies 4-7.

There are other cell types where intranuclear rim localization of actin has been reported. In starfish oocytes, an F-actin shell forms at the nuclear envelope to promote nuclear envelope breakdown (PMID 24909322, 29350616, 31989921). Similar F-actin nuclear shells have been observed in a variety of other species including early sea urchin and sand dollar embryos, *Nematostella vectensis*, and polychaete worms (PMID 17685442, 25433655, 10213079). F-actin is also observed at the nuclear rim in *Xenopus laevis* oocytes (PMID 16489345) and mouse oocytes (PMID 35112707) as well as in mesenchymal stem cells (PMID 31648392) and several cultured mammalian cell lines (PMID 23558171). These observations support the idea that the intranuclear rim of actin we observe is not an artifact of the *Xenopus* extract system. Rather, the prominence of this structure may depend on cell type, species, developmental stage, and/or disease state. For example, accumulation of actin at the intranuclear rim may be a characteristic of nuclei in oocytes and early stage embryos. That could explain why we observe this actin structure in egg extract, indicating the extract system is recapitulating a normal feature of oocyte nuclei. We argue that this actin structure is indeed physiologically relevant, at least in the context of early development,

© 2022. Published by The Company of Biologists under the terms of the Creative Commons Attribution License (https://creativecommons.org/licenses/by/4.0/).

underscoring the need to examine this structure in other cell types. We now comment on this idea in the manuscript.

While nuclear F-actin appears rod-like in many published studies, there are also examples where some accumulation of actin at the nuclear rim is evident. Examples include:
Baarlink et al. (PMID 29131140) - Fig. 1A (time point 21:00), Fig. 2A, Fig. 3A (time point 27:30), Fig. 4A (later time points), Fig. 6F, Fig. S6E
Lamm et al. (PMID 33257806) - Fig. S1J (0:05), Fig. S1K (nucleus on the far right), Fig. S3H (nucleus on the far right).

Minor points:

10.) It would be interesting to visualize lamin A/C in the experiments performed in Fig 4. Does it localize to the nuclear envelope? Does this show a similarly uneven distribution as NPCs and lamin B?

We have performed this experiment and do observe lamin A localized to the nuclear envelope. These data are now included in Fig. S3C and mentioned in the text.

11) figure legend 5. Although the figure is largely self-explanatory the sentence “(A) Representative images are shown for overexpression of nuclear targeted actin and/or lamin A knockdown” is formally not correct as the panel also includes pictures showing control RNAi and mCherry only. Same applies to (C).

We have revised these figure legends.

12.) I suggest switching panel C/D and panel E/F in figure 5 to follow the flow of the text.

We corrected the panel order.

13.) Given that fertilized Xenopus eggs lack lamin A/C it remains open what in the first embryonic cell cycles prevents nuclear actin from generating bi-lobuled nuclei in the natural situation. In other words, what takes the function of cytochalasin B in the early developing tadpoles? This might be worth to discuss or address in future studies.

This is a very interesting point. We now include some discussion of this issue in the text. Possible explanations include: 1) fast cell cycles with short interphases do not allow time for nuclear bilobes to form, 2) factors other than lamin A stabilize nuclei against blebs, 3) the presence of a cell cortex (absent from extracts) and cytoskeletal connections between the nucleus and cell cortex stabilize nuclei from blebbing, 4) increased exportin-6 expression after fertilization (PMID 26555057).

Second decision letter

MS ID#: JOCES/2021/259692

MS TITLE: Nuclear F-actin and Lamin A antagonistically modulate nuclear shape

AUTHORS: Sampada Mishra and Daniel L. Levy
ARTICLE TYPE: Research Article

We have now reached a decision on the above manuscript.

To see the reviewers' reports and a copy of this decision letter, please go to: https://submit-jcs.biologists.org and click on the 'Manuscripts with Decisions' queue in the Author Area. (Corresponding author only has access to reviews.)

© 2022. Published by The Company of Biologists under the terms of the Creative Commons Attribution License (https://creativecommons.org/licenses/by/4.0/).

As you will see, while reviewer #2 recognizes that most initial concerns have been addressed in your revised manuscript, reviewer #1 still raised criticisms that will require amendments to your manuscript. As I understand further experiments are difficult to include at this stage, I suggest to tone down the conclusions, mainly in the abstract, acknowledging that the mechanism observed in Xenopus might not be essential for HeLa cells since it is only observed in a subset of cells. I hope that you will be able to carry these revisions because I would like to be able to accept your paper.

Please ensure that you clearly highlight all changes made in the revised manuscript. Please avoid using 'Tracked changes' in Word files as these are lost in PDF conversion.

I should be grateful if you would also provide a point-by-point response detailing how you have dealt with the points raised by the reviewers in the 'Response to Reviewers' box. Please attend to all of the reviewers' comments. If you do not agree with any of their criticisms or suggestions please explain clearly why this is so.

Reviewer 1

Advance summary and potential significance to field

In the reviewed version of the manuscript entitled “Nuclear F-actin and Lamin A antagonistically modulate nuclear shape” the authors addressed most of the main concerns previously raised by the reviewer. Nevertheless, the point of novelty and the new data on the observation of actin at the nuclear rim in HeLa cells is still not convincing, as described below. Regarding my first comment on the “The findings described using HeLa cells are not novel and were described in Lamm et al NCB 2020 and Baarlink et al NCB 2017.” I meant the finding described in this manuscript using HeLa cells. I apologise for the confusion. Nevertheless, these published papers remove some of the novelty of the current paper regarding Lamin A/C.

Regarding the question “is the nuclear cortical actin observed in the Xenopus extracts also occurring in the nucleus of cells?”, the new data presented by the authors does not suggest the existence of nuclear cortical actin in HeLa cells.
Movie 4-5 and 6-7: both movies correspond to cell undergoing mitosis, and the only frame where it looks like we observe actin in the nucleus rim, are very similar to cortical actin at the plasma membrane during mitosis. Can the authors clarify? Also, in movies 7-6 the reviewer cannot observe any accumulation of actin at the nuclear rim. The images provided in sup fig 6B are more convincing but nevertheless they look like actin cable bundles that are curved in the periphery of the nucleus, and in this way very different from the actin rim in xenopus. Finally, the examples from Baarlink et al and Lamm et all mentioned by the author do not look like actin rims showed in Xenopus, nor like actin at the nucleus periphery in sup fig 6B.
The authors also discuss the observation of cortical actin in oocytes from different species and the authors suggest that this structure might depend on the cell type and developmental stage. Since oocyte nuclei and early embryonic stage cells are much bigger, this could explain the existence of such mechanism in large nuclei, and therefore does not occur (or it is not so well observed) in differentiated cells such as HeLa cells, which are much smaller.

Regarding the asymmetry of NPC complex and LaminB3, observed in Xenopus extracts but not in HeLa cells, the authors provide one image of asymmetric localization of NPC and LaminB3, and described it as a “subset” of cells. What is the percentage of cells that these phenotype is observed? And how can this be relevant for the proposed mechanism?

Regarding the next point where the reviewer stated: “Therefore, all these observations raise the possibility that the observed role of F-actin in the nucleus is specific to the used in vitro system, and not occurring in the nuclei of cells. It is therefore important that the authors provide a rational and experimental evidence supporting that the in vitro system they are using with f-
actin inside the nucleus mimics what is observed in cells”. The answer of the reviewers is based on a subset of observations, without a solid quantification.
Such quantification is required to claim tha the mechanism described in this paper is also observed in HeLa cells. Alternatively, the authors should tone down their conclusions, mainly in the abstract

© 2022. Published by The Company of Biologists under the terms of the Creative Commons Attribution License (https://creativecommons.org/licenses/by/4.0/).

to suggest that such mechanism observed in Xenopus might not be of great importance for HeLa cells since it is only observed in a subset of cells.

Comments for the author

Regarding my first comment on the “The findings described using HeLa cells are not novel and were described in Lamm et al NCB 2020 and Baarlink et al NCB 2017.” I meant the finding described in this manuscript using HeLa cells. I apologise for the confusion. Nevertheless, these published papers remove some of the novelty of the current paper regarding Lamin A/C.
Regarding the question “is the nuclear cortical actin observed in the Xenopus extracts also occurring in the nucleus of cells?”, the new data presented by the authors does not suggest the existence of nuclear cortical actin in HeLa cells.
Movie 4-5 and 6-7: both movies correspond to cell undergoing mitosis, and the only frame where it looks like we observe actin in the nucleus rim, are very similar to cortical actin at the plasma membrane during mitosis. Can the authors clarify? Also, in movies 7-6 the reviewer cannot observe any accumulation of actin at the nuclear rim. The images provided in sup fig 6B are more convincing but nevertheless they look like actin cable bundles that are curved in the periphery of the nucleus, and in this way very different from the actin rim in xenopus. Finally, the examples from Baarlink et al and Lamm et all mentioned by the author do not look like actin rims showed in Xenopus, nor like actin at the nucleus periphery in sup fig 6B.
The authors also discuss the observation of cortical actin in oocytes from different species and the authors suggest that this structure might depend on the cell type and developmental stage. Since oocyte nuclei and early embryonic stage cells are much bigger, this could explain the existence of such mechanism in large nuclei, and therefore does not occur (or it is not so well observed) in differentiated cells such as HeLa cells, which are much smaller.
Regarding the asymmetry of NPC complex and LaminB3, observed in Xenopus extracts but not in HeLa cells, the authors provide one image of asymmetric localization of NPC and LaminB3, and described it as a “subset” of cells. What is the percentage of cells that these phenotype is observed? And how can this be relevant for the proposed mechanism?
Regarding the next point where the reviewer stated: “Therefore, all these observations raise the possibility that the observed role of F-actin in the nucleus is specific to the used in vitro system, and not occurring in the nuclei of cells. It is therefore important that the authors provide a rational and experimental evidence supporting that the in vitro system they are using with f-
actin inside the nucleus mimics what is observed in cells”. The answer of the reviewers is based on a subset of observations, without a solid quantification.
Such quantification is required to claim tha the mechanism described in this paper is also observed in HeLa cells. Alternatively, the authors should tone down their conclusions, mainly in the abstract to suggest that such mechanism observed in Xenopus might not be of great importance for HeLa cells since it is only observed in a subset of cells.

<u>Reviewer 2</u>

Advance summary and potential significance to field

the authors have addressed the points raised by the reviewers.

Comments for the author

the authors have addressed the points raised by the reviewers.

© 2022. Published by The Company of Biologists under the terms of the Creative Commons Attribution License (https://creativecommons.org/licenses/by/4.0/).

Second revision

Author response to reviewers' comments

Response to reviews

Reviewer comments are copied in *italics*. Our responses follow below each reviewer comment.

Editor

As you will see, while reviewer #2 recognizes that most initial concerns have been addressed in your revised manuscript, reviewer #1 still raised criticisms that will require amendments to your manuscript. As I understand further experiments are difficult to include at this stage, I suggest to tone down the conclusions, mainly in the abstract, acknowledging that the mechanism observed in Xenopus might not be essential for HeLa cells since it is only observed in a subset of cells. I hope that you will be able to carry these revisions because I would like to be able to accept your paper.

We have addressed the comments below by modifying the text and movies. In particular, we have toned down the conclusion in the abstract by indicating that the underlying mechanisms may differ in *Xenopus* egg extract versus HeLa cells.

Reviewer 1

In the reviewed version of the manuscript entitled "Nuclear F-actin and Lamin A antagonistically modulate nuclear shape" the authors addressed most of the main concerns previously raised by the reviewer. Nevertheless, the point of novelty and the new data on the observation of actin at the nuclear rim in HeLa cells is still not convincing, as described below. Regarding my first comment on the "The findings described using HeLa cells are not novel and were described in Lamm et al NCB 2020 and Baarlink et al NCB 2017." I meant the finding described in this manuscript using HeLa cells. I apologise for the confusion. Nevertheless, these published papers remove some of the novelty of the current paper regarding Lamin A/C.

As we noted previously, neither of these papers examine nuclear shape. In addition, neither of these papers study the interplay between Lamin A/C and nuclear actin to the extent reported in our manuscript. Thus we still argue that there is novelty in our HeLa studies.

Regarding the question "is the nuclear cortical actin observed in the Xenopus extracts also occurring in the nucleus of cells?", the new data presented by the authors does not suggest the existence of nuclear cortical actin in HeLa cells. Movie 4-5 and 6-7: both movies correspond to cell undergoing mitosis, and the only frame where it looks like we observe actin in the nucleus rim, are very similar to cortical actin at the plasma membrane during mitosis. Can the authors clarify? Also, in movies 7-6 the reviewer cannot observe any accumulation of actin at the nuclear rim.

The reviewer is correct that one frame in Movies 4-5 shows mitosis. We now note in the movie legends the frame corresponding to mitosis. Movies 6-7 only show interphase, and we now note this in the movie legends. As mentioned in the movie legends, nuclear actin probe nAC-GFP is being visualized. Since almost all frames in these movies show interphase nuclei, the observed signal corresponds to actin within the nucleus and not in the cytoplasm or at the plasma membrane. This is something we validated by co- staining for DNA and lamin B1 (Fig. S6B), and it is also evident that the vast majority of the nAC-GFP signal is nuclear when brightness/contrast are enhanced. To clarify, we have added arrows to the movies to highlight areas where we see an enrichment of nuclear actin at the nuclear rim. In Movie 6, nearly the entire nuclear rim is circumscribed by actin.

The images provided in sup fig 6B are more convincing, but nevertheless they look like actin cable bundles that are curved in the periphery of the nucleus, and in this way very different from the actin rim in xenopus. Finally, the examples from Baarlink et al and Lamm et all mentioned by the author do not look like actin rims showed in Xenopus, nor like actin at the nucleus periphery in sup fig 6B.

In describing Fig. S6B we now note that the rim-localized actin may correspond to actin cable bundles curving along the periphery of the nucleus.

© 2022. Published by The Company of Biologists under the terms of the Creative Commons Attribution License (https://creativecommons.org/licenses/by/4.0/).

The authors also discuss the observation of cortical actin in oocytes from different species and the authors suggest that this structure might depend on the cell type and developmental stage. Since oocyte nuclei and early embryonic stage cells are much bigger, this could explain the existence of such mechanism in large nuclei, and therefore does not occur (or it is not so well observed) in differentiated cells such as HeLa cells, which are much smaller.

We have now added this point to the discussion.

Regarding the asymmetry of NPC complex and LaminB3, observed in Xenopus extracts but not in HeLa cells, the authors provide one image of asymmetric localization of NPC and LaminB3, and described it as a "subset" of cells. What is the percentage of cells that these phenotype is observed? And how can this be relevant for the proposed mechanism?

The reviewer is referring to Fig. S6A. We note in the text and figure legend that this phenotype was observed in 59.7%±7.1% (mean±SD) of nuclei with nuclear circularity below 0.6, based on 3 independent experiments and analysis of 51 nuclei. As far as the relevance of this observation to the proposed mechanism, we have a paragraph in the Discussion devoted to this topic: "Misshapen nuclei formed in *Xenopus* extract and HeLa cells frequently exhibited uneven distributions of NE proteins, for instance regions of reduced DNA density often correlated with reduced staining for NPCs and lamins, reminiscent of nuclear blebs that form during confined cell migration (Denais et al., 2016; Raab et al., 2016). Similarly, Lamin A knockdown in MEFs resulted in elongated nuclei with reduced Lamin B, Lap2, and NPCs at one pole (Sullivan et al., 1999), and nuclear blebs in Lamin A-mutated progeroid fibroblasts exhibited reduced staining for DNA, NPCs, and Lamin B1 (Bercht Pfleghaar et al., 2015). What might account for this uneven distribution of lamins and NPCs across the NE? LEM-domain proteins like Lap2, Emerin, and Man1 interact with both the nuclear lamina and BAF-bound DNA, effectively tethering chromatin to the lamina (Shimi et al., 2004; Wilson and Foisner, 2010). Similarly, the nucleoporin ELYS binds chromatin via histone interactions (Zierhut et al., 2014). Lamins and NPCs might therefore be preferentially tethered to regions with the most DNA through chromatin-mediated interactions. Another intriguing possibility is that upon nuclear import, lamins are preferentially incorporated into the region of lamina close to the importing NPC. This might explain why NE regions containing fewer NPCs also show reduced lamin incorporation. Since the nuclear lobe without DNA grows more rapidly than the DNA- containing lobe in F-actin intact extracts, this system can be used to study uncoupling of nuclear growth and chromatin dynamics."

Regarding the next point where the reviewer stated: "Therefore, all these observations raise the possibility that the observed role of F-actin in the nucleus is specific to the used in vitro system, and not occurring in the nuclei of cells. It is therefore important that the authors provide a rational and experimental evidence supporting that the in vitro system they are using with f-actin inside the nucleus mimics what is observed in cells". The answer of the reviewers is based on a subset of observations, without a solid quantification. Such quantification is required to claim tha the mechanism described in this paper is also observed in HeLa cells. Alternatively, the authors should tone down their conclusions, mainly in the abstract to suggest that such mechanism observed in Xenopus might not be of great importance for HeLa cells since it is only observed in a subset of cells.

We would argue that we have included appropriate quantifications for the nuclear F- actin we observe in HeLa cells, for example see the figure legend for Fig. S6.
Nonetheless, we have modified the abstract to indicate that the underlying mechanisms may differ in *Xenopus* egg extract versus HeLa cells. We have also included this point in the Discussion.

Reviewer 2

The authors have addressed the points raised by the reviewers.

We appreciate the reviewer's support.

© 2022. Published by The Company of Biologists under the terms of the Creative Commons Attribution License (https://creativecommons.org/licenses/by/4.0/).

Third decision letter

MS ID#: JOCES/2021/259692

MS TITLE: Nuclear F-actin and Lamin A antagonistically modulate nuclear shape

AUTHORS: Sampada Mishra and Daniel L. Levy
ARTICLE TYPE: Research Article

I am happy to tell you that your manuscript has been accepted for publication in Journal of Cell Science, pending standard ethics checks.

© 2022. Published by The Company of Biologists under the terms of the Creative Commons Attribution License (https://creativecommons.org/licenses/by/4.0/).
